# iSCORE-PD: an isogenic stem cell collection to research Parkinson’s Disease

**DOI:** 10.1101/2024.02.12.579917

**Published:** 2024-02-13

**Authors:** Oriol Busquets, Hanqin Li, Khaja Mohieddin Syed, Pilar Alvarez Jerez, Jesse Dunnack, Riana Lo Bu, Yogendra Verma, Gabriella R. Pangilinan, Annika Martin, Jannes Straub, YuXin Du, Vivien M. Simon, Steven Poser, Zipporiah Bush, Jessica Diaz, Atehsa Sahagun, Jianpu Gao, Dena G. Hernandez, Kristin S. Levine, Ezgi O. Booth, Helen S. Bateup, Donald C. Rio, Dirk Hockemeyer, Cornelis Blauwendraat, Frank Soldner

**Affiliations:** 1 Dominick P. Purpura Department of Neuroscience, Albert Einstein College of Medicine, Rose F. Kennedy Center, Albert Einstein College of Medicine, 1410 Pelham Parkway South, Bronx, NY 10461, USA.; 2 Ruth L. and David S. Gottesman Institute for Stem Cell and Regenerative Medicine Research, Albert Einstein College of Medicine, 1301 Morris Park Ave., Bronx, NY 10461, USA.; 3 Aligning Science Across Parkinson’s (ASAP) Collaborative Research Network, Chevy Chase, MD, 20815, USA.; 4 Department of Molecular and Cell Biology, University of California, Berkeley, Berkeley, CA 94720, USA.; 5 Innovative Genomics Institute, University of California, Berkeley, CA, 94720, USA.; 6 Center for Alzheimer’s and Related Dementias, National Institute on Aging and National Institute of Neurological Disorders and Stroke, National Institutes of Health, Bethesda, MD, 20892, USA.; 7 Department of Neurodegenerative Disease, UCL Queen Square Institute of Neurology, University College London, London, UK.; 8 Department of Genetics, Albert Einstein College of Medicine, 1301 Morris Park Ave., Bronx, NY 10461, USA.; 9 Chan Zuckerberg Biohub, San Francisco, CA, 94158, USA.; 10 Helen Wills Neuroscience Institute, University of California, Berkeley, Berkeley, CA 94720, USA.; 11 Laboratory of Neurogenetics, National Institute on Aging, National Institutes of Health, Bethesda, MD, 20892, USA.; 12 These authors contributed equally.; 13 Lead contact

## Abstract

Parkinson’s disease (PD) is a neurodegenerative disorder caused by complex genetic and environmental factors. Genome-edited human pluripotent stem cells (hPSCs) offer the uniique potential to advance our understanding of PD etiology by providing disease-relevant cell-types carrying patient mutations along with isogenic control cells. To facilitate this experimental approach, we generated a collection of 55 cell lines genetically engineered to harbor mutations in genes associated with monogenic PD (*SNCA* A53T, *SNCA* A30P, *PRKN* Ex3del, *PINK1* Q129X, *DJ1/PARK7* Ex1–5del, *LRRK2* G2019S, *ATP13A2* FS, *FBXO7* R498X/FS, *DNAJC6* c.801 A>G+FS, *SYNJ1* R258Q/FS, *VPS13C* A444P, *VPS13C* W395C, *GBA1* IVS2+1). All mutations were generated in a fully characterized and sequenced female human embryonic stem cell (hESC) line (WIBR3; NIH approval number NIHhESC-10–0079) using CRISPR/Cas9 or prime editing-based approaches. We implemented rigorous quality controls, including high density genotyping to detect structural variants and confirm the genomic integrity of each cell line. This systematic approach ensures the high quality of our stem cell collection, highlights differences between conventional CRISPR/Cas9 and prime editing and provides a roadmap for how to generate gene-edited hPSCs collections at scale in an academic setting. We expect that our isogenic stem cell collection will become an accessible platform for the study of PD, which can be used by investigators to understand the molecular pathophysiology of PD in a human cellular setting.

## Introduction

Parkinson’s disease (PD) is the second most common chronic neurodegenerative disorder with a prevalence of more than 1% in the population over the age of 60^1^. PD is primarily characterized by a progressive loss of dopaminergic neurons in the midbrain and in most cases the presence of proteinaceous inclusions (Lewy bodies) in affected cells^2–5^. However, neurodegeneration and PD pathology is highly variable and widespread throughout the brain^6^. Furthermore, it is increasingly recognized that non-neuronal cell types, including astrocytes, oligodendrocytes and microglia play important roles in the pathogenesis of the disease^7^. The precise etiology leading to neuronal cell loss is largely unknown. However, the discovery of mutations in more than 20 genes linked to rare monogenic forms of PD revealed a broad spectrum of molecular and cellular pathways that likely contribute to PD pathology including vesicle transport, lysosomal function, mitochondrial function, and ER protein folding^4,8^. Notably, individuals with PD who carry these mutations can present with highly heterogeneous clinical and pathological features. These can include incomplete penetrance, variable age of onset and highly diverse patterns of Lewy body pathology; in some cases, this classical hallmark of the disease is completely absent^6^. This variability has been described within the same mutation and across different mutations associated with PD^5,9^. Recognizing this complexity of PD, it is widely acknowledged that additional genetic and environmental modifiers play significant roles in the pathophysiology even for monogenic forms of the disease^4^. Therefore, it remains challenging to distinguish common versus mutation-specific phenotypic features, and to gain a deeper understanding of how the diverse cellular pathways linked to PD-risk genes are connected and perturbed in PD.

Genome editing of human pluripotent stem cells (hPSCs), encompassing human embryonic stem cells (hESCs) and induced pluripotent stem cells (hiPSCs), is increasingly utilized to establish isogenic cellular models for human diseases such as PD^10–29^. This approach involves the use of genome editing technologies such as CRISPR/Cas9 or prime editing systems to generate isogenic cell lines by either genetically correcting or inserting disease-linked mutations in hPSCs. This system has the key advantage of analyzing the effect of a mutation in the same genetic background. The phenotypic characterization of such isogenic pairs of cells, comparing cells with and without PD-associated mutations, have provided significant insights into the molecular mechanisms underlying familial forms of PD^10–29^. However, as each isogenic pair is generated on a distinct, patient-specific genetic background, conducting a cross-comparison between various PD mutations remains challenging due to the confounding effects of genetic modifier loci inherent to the genetic background of each individual^10,30^.

As a consequence, we still lack a unified genetic interaction map for how different monogenic PD-related genes, their pathogenic mutations and their respective phenotypes intersect to drive PD-pathology. Ongoing initiatives across different diseases are underway to streamline the development of isogenic disease-relevant hPSC collections in a common, thoroughly characterized parental hPSC line^30^. Here, we report the generation of such a resource for PD as part of the Aligning Science Across Parkinson’s (ASAP) research network, which we have termed iSCORE-PD (Isogenic Stem Cell Collection to Research Parkinson’s Disease). We used state-of-the-art CRISPR/Cas9 and prime editing approaches to establish a total of 55 clonal cell lines carrying disease-causing or high-risk PD-associated mutations in 11 genes (*SNCA*, *PRKN*, *PINK1*, *DJ1/PARK7*, *LRRK2*, *ATP13A2*, *FBXO7*, *DNAJC6*, *SYNJ1*, *VPS13C*, and *GBA1*) along with isogenic control lines. All cell lines were derived from a fully characterized and sequenced female hESC (WIBR3; NIH approval number NIHhESC-10–0079)^31^ and subjected to rigorous quality controls. This comprehensive collection of isogenic hPSCs is accessible to the community to enable the cross-comparison of disease-related phenotypes and accelerate PD research.

## Results

### Characterization of the hESC line WIBR3.

**Figure 1.**
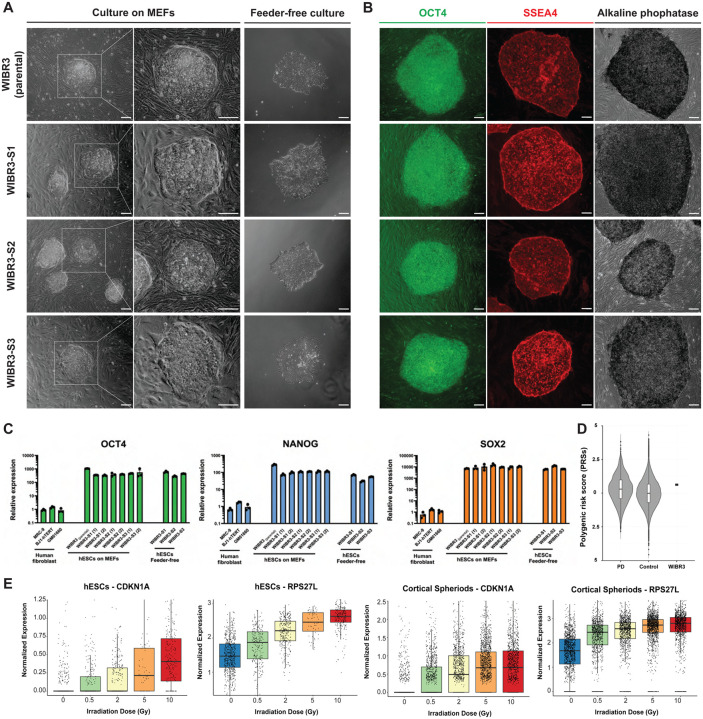
WIBR3 hESC cell line characterization. (A) Phase contrast images of parental WIBR3 hESCs and subclones WIBR3-S1, WIBR3-S2 and WIBR3-S3 cultured onto MEFs and in feeder-free conditions. Scale bar 100 μm. (B) Immunocytochemistry for pluripotency markers OCT4 (green) and SSEA4 (red) and staining for alkaline phosphatase (black) of WIBR3 (parental) hESCs and subclones WIBR3-S1, WIBR3-S2 and WIBR3-S3 cultured onto MEFs. Scale bar 100 μm. (C) qRT-PCR analysis for the relative expression of pluripotency markers OCT4, NANOG and SOX2 in human primary fibroblasts (MRC-9, BJ1-hTERT and GM01660), WIBR3 (parental) hESCs and subclones WIBR3-S1, WIBR3-S2 and WIBR3-S3 hESCs cultured on MEFs and in feeder-free conditions. Relative expression levels were normalized to expression of these genes in primary fibroblasts. (1) and (2) indicate independent samples. (N=3; Mean +/− SEM). (D) Polygenic risk scores (PRSs) for PD comparing WIBR3 hESCs to population-centered Z score distribution for PD PRSs in individuals with PD and the normal population from the UK Biobank. (E) Assessment of p53 pathway activity following irradiation (0.5, 2, 5 and 10 Gy) of WIBR3 (parental) hESCs (1464 cells) and WIBR3-derived cortical spheroids (5920 cells) by scRNA-seq analysis for the expression of DNA damage response genes CDKN1A and RPS27L ( box plot showing interquartile intervals with a line at the median).

A major goal of the work described in this publication is to complement ongoing initiatives to establish comprehensive collections of hPSC lines carrying mutations associated with PD and related neurodegenerative diseases^30^. The aim of the approach reported here is to eliminate the genetic variability among cell lines in order to highlight disease-relevant pathophysiological signatures. One example of these efforts is the recently described iPSC Neurodegenerative Disease Initiative (iNDI) from the NIH’s Center for Alzheimer’s and Related Dementias (CARD), which utilized the KOLF2.1J hiPSC line^30^. This cell line, derived from a male donor, is currently recognized as a benchmark reference for neurodegenerative disease research to facilitate the comparison of disease-associated phenotypes across different laboratories. Although hiPSCs have proven instrumental for disease modeling, concerns remain regarding any genetic alterations present in the somatic donor cells before reprogramming, reprogramming-induced genetic alterations, incomplete epigenetic reprogramming, and aberrant genomic imprinting^10,32,33^. Given these considerations, and the necessity for incorporating cells from both sexes, we opted to use the female hESC line WIBR3 of European descent (NIH approval number NIHhESC-10–0079). This cell line has been previously demonstrated to maintain a stable karyotype over prolonged *in vitro* culture and has been widely used to model human diseases including PD^29,31,34–40^. For our effort, we acquired early passage WIBR3 cells (P19) and initially generated 3 independent single cell-derived subclones (WIBR3-S1, WIBR3-S2, WIBR3-S3). The parental line and its subclones showed regular growth and morphology when cultured on mouse fibroblast feeders (MEFs) and under feeder free conditions in mTeSR^™^ Plus media (Figure 1A). We validated the pluripotency of all cell lines through the detection of pluripotency markers using immunocytochemistry and qRT-PCR (Figure 1B,C; Supplemental Figure 1). Furthermore, we analyzed the genomic integrity of all cell lines using standard array comparative genomic hybridization (aCGH) and a modified high density Illumina Infinium Global Diversity Array (GDA) Neuro booster Array (NBA). This analysis confirmed a normal karyotype and the absence of larger structural alterations (> ~500 kb) for both the parental WIBR3 line and its derived sub-clones (complete karyotype data is available at https://www.amp-pd.org/).

To determine the presence of insertions and deletions (indels) at higher resolution and to identify potential pathogenic single nucleotide variants (SNVs) in the parental WIBR3 hESC line, we performed long-read whole genome sequencing (WGS) combining Pacific Biosciences (PacBio, average coverage 28.39X, median read length 18 kb) and Oxford Nanopore Technologies (Nanopore, average coverage 43X, median read length 84 kb). The initial analysis for structural variants, using the Truvari algorithm to integrate the PacBio and Nanopore datasets, identified a total of 20,561 high confident structural variants in the WIBR3 parental line compared to the reference human genome [GRCh38/hg38] (Supplemental Table 1). Among these, 109 were localized to coding exons, impacting 102 genes (Supplemental Table 1). The number and distribution of these structural variants is comparable to those observed in the general human population^41,42^. As our goal is to model PD and related neurodegenerative diseases, we determined that none of these structural variants affects genes with known pathogenic mutations in PD, Alzheimer’s diseases (AD) and AD-related dementias (ADRD), or risk genes identified in GWAS associated with these diseases^4,43–45^ (Supplemental Table 1). Additionally, we annotated the integrated structural variant calls using SVAnna to determine if any variant was of high priority for the phenotype terms HP:0002180 (neurodegeneration) and HP:0000707 (abnormality of the nervous system). SVAnna did not determine any structural variant as high priority for either of these terms.

Next, we identified the number and distribution of coding, missense, frameshift and predicted loss of function (LOF) SNVs in the parental WIBR3 cell line compared to the reference human genome [GRCh38/hg38] (Supplemental Figure 1B). This analysis identified 6613 missense SNVs in 3933 coding genes and 120 potential loss of function mutations (including 15 startloss, 54 stopgain, 13 stoploss, 18 frameshift deletion, and 20 frameshift insertion). A full list of variants can be found in Supplemental Table 2 and the full genome is available for broad data sharing (https://www.amp-pd.org/ via GP2 data sharing agreement). Overall, the number and distribution of these variants is comparable to other sequenced hPSC lines^30^ and to the human population in the gnomAD database^46,47^.

To determine the presence of potentially pathogenic variants in the parental WIBR3 cell line, we annotate all SNVs using ClinVar. Collectively, these analyses revealed 48 variants that are listed as pathogenic or conflicting interpretations of pathogenicity, however none of these are in a neurological phenotype of interest or are convincingly pathogenic (Supplemental Table 2). As we aim to provide our cell collection to study PD and related neurodegenerative diseases, we analyzed the WGS to calculate the polygenic risk scores (PRS) based on the cumulative number of GWAS risk variants for PD (Figure 1D)^43^. The analysis indicated that the PRS of the parental WIBR3 falls within the range observed in the normal population. Next, we specifically focused on identifying high risk variants in known neurodegenerative diseases-associated genes including APOE, TMEM106B and MAPT. This analysis determined that WIBR3 is heterozygous for the APOE ε4 allele which is a risk factor for Alzheimer’s disease, heterozygous for rs3173615 (TMEM106B p.T185S)^48^ which has been reported to be a modifier of frontotemporal dementia and homozygous for the MAPT H1 allele which is a gene of interest in several diseases^49,50^.

It is widely recognized that hPSCs can accumulate genetic alterations over time that provide a growth advantage in cell culture. Notably, mutations in the p53 tumor suppressor pathway have been frequently observed in various hPSC lines^51–53^. To evaluate the function of the p53 pathway in the parental WIBR3 cell line, we analyzed the p53-dependent DNA damage response following irradiation (Figure 1E). This analysis confirmed a robust p53 response in undifferentiated cells and *in vitro*-derived cortical spheroids (Figure 1E).

### WIBR3 cells differentiate into PD-relevant cell types.

**Figure 2.**
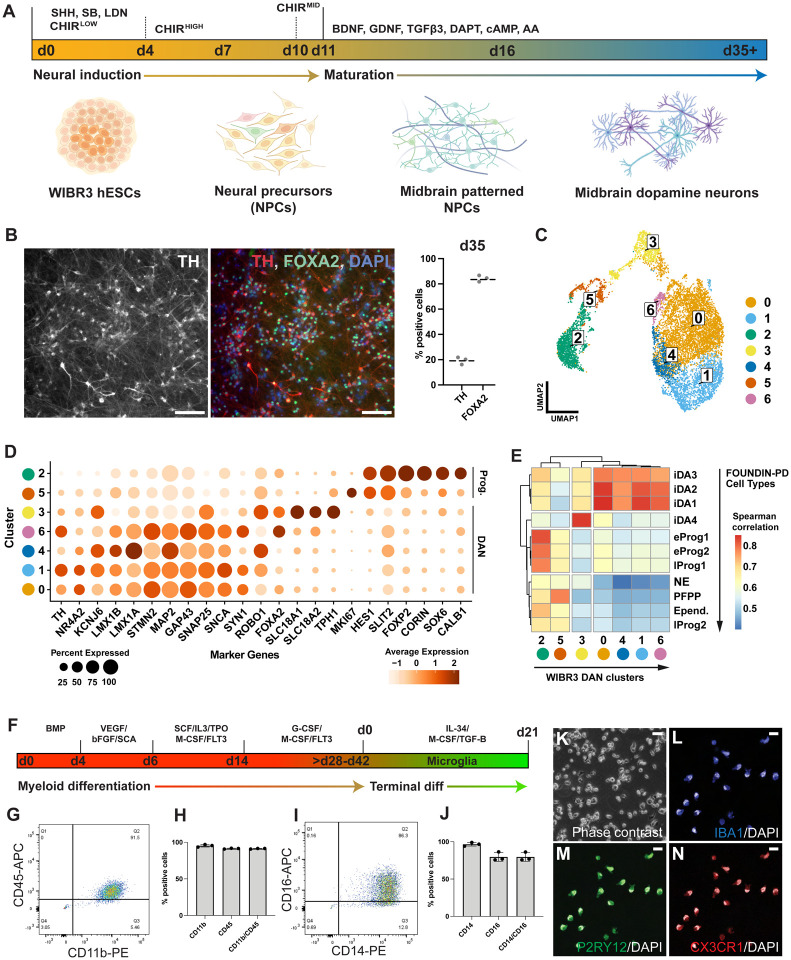
WIBR3 differentiation potential into dopaminergic neurons and microglia subtypes in 2D culture. (A) Schematic depicting the protocol for in vitro differentiation of dopaminergic neurons from WIBR3 hESCs. (B) Immunocytochemistry and quantification of TH and FOXA2 expressing cells in WIBR3 (parental) hESC-derived dopaminergic neurons at day 35. Scale bar 100 μm.(N=3). (C) Uniform manifold approximation and projection (UMAP) plot of scRNA-Seq analysis at day 35 to 37 of dopaminergic neuron differentiation from WIBR3-S1, WIBR3-S2 and WIBR3-S3 hESCs showing 10,097 cells separated into 7 coarse clusters. (D) Dot plot showing expression of key midbrain progenitor and dopamine neuron marker genes across different cluster identities indicates that clusters identified in (C) represent midbrain progenitors and dopaminergic neurons at different developmental stages. Dot size indicates the proportion of cells in a cluster expressing a given gene, while color intensity indicates its average expression. (E) Heatmap depicting Spearman correlation coefficients between pseudo-bulk expression profiles of 7 WIBR3 clusters identified in (C) compared to pseudo-bulk expression profiles of 11 FOUNDIN-PD cell types. (F) Schematic depicting in vitro microglia differentiation protocol from WIBR3 hESCs. (G–J) Representative flow cytometry (FACS) analysis (G,I) and quantification (H,J) of CD14/CD16 and CD11b/CD45 expression in hESC-derived iMPs from subclones WIBR3-S1, WIBR3-S2 and WIBR3-S3. (K–N) Representative phase contrast (K, Scale bar 50 μm) and immunostaining (L–N, Scale bar 10 μm) images of in vitro differentiated microglia derived from subclone WIBR3-S1 for microglia-specific markers IBA1 (blue), P2RY12 (green)) and CX3CR1 (red) (terminal differentiation day 14).

As PD is characterized by the chronic progressive loss of dopaminergic (DA) neurons in the substantia nigra, the effective generation of these cell types is crucial for *in vitro* modeling of PD. Therefore, we implemented a previously established protocol^54,55^ to differentiate the three independently derived WIBR3 subclones (WIBR3-S1, WIBR3-S2, WIBR3-S3) into midbrain-specified dopaminergic neurons. Briefly, WIBR3 hESCs were subjected to dual SMAD inhibition for neural induction along with sonic hedgehog (SHH) agonist exposure for midbrain specific patterning for 11 days. Concentrations of the GSK-3 inhibitor (CHIR99021) were increased from days 4 to 11. From day 11, committed midbrain neural progenitors were differentiated into dopamine neurons until day 35 (Figure 2A). For each subclone, we determined the efficacy of neural induction into neural precursor cells and dopaminergic neurons by analyzing the expression of midbrain-specific genes. At early time points (day 11 and day 25), the *in vitro* differentiated cultures expressed the expected midbrain floor plate markers at levels comparable to those in concurrently differentiated KOLF2.1J hiPSCs (Supplemental Figure 2B). At day 35 of differentiation, greater than 80% of cells expressed FOXA2, a marker for early midbrain floorplate neuronal precursors, and approximately 20% expressed the dopaminergic neuron marker tyrosine hydroxylase (TH), indicating the generation of midbrain-specified dopaminergic neurons (Figure 2B; Supplemental Figure 2A).

To further characterize the DA neuron cultures, we performed single-cell RNA sequencing (scRNA-seq) at day 35–37 post-differentiation from hESCs. We profiled a total of 10,097 cells across the three independently assayed subclones, yielding an aggregate dataset. Following standard approaches for dimensionality reduction and unsupervised clustering, seven distinct clusters were present in the integrated dataset, each composed of cells belonging to all subclones (Figure 2C, Supplemental Figure 3A) Out of the seven clusters, clusters 0, 1, 4, 6 and 3 expressed canonical dopaminergic neuron markers (*KCNJ6, TH, NR4A2*), whereas clusters 2 and 5 expressed midbrain progenitor markers (*CALB1, SOX6 and CORIN*) (Figure 2D, Supplemental Figure 3B–D). To further compare the differentiation propensity of WIBR3 cells against other cell lines with different genetic backgrounds, we compared our dataset with the recently published Foundational Data Initiative for Parkinson’s Disease (FOUNDIN-PD) data (Figure 2E)^56^. The FOUNDIN-PD dataset includes single-cell RNA-seq data from midbrain DA neuron cultures at day 65, derived from 80 distinct iPSC lines using a comparable *in vitro* differentiation protocol^56^. This comparison revealed that the clusters representing DA neuron populations (clusters 0, 1, 4, 6, and 3) in our dataset showed the highest Spearman correlation scores with iDA1, iDA2, iDA3 and iDA4 neuron clusters identified in the FOUNDIN-PD data and clusters 2 and 5 from our dataset have higher similarity to progenitor populations (Supplemental Figure 4A–D). Together, this analysis indicates a high similarity in the expression profiles of our WIBR3-derived cell types to the midbrain-derived cell types found in the FOUNDIN-PD dataset; currently considered the gold standard for *in vitro*-derived midbrain-specific DA neurons. This is relevant as it should allow the integration of data generated from the iSCORE-PD collection with the FOUNDIN-PD datasets.

Recent evidence suggests that the impact of PD-associated mutations extends beyond neurons, with effects on cell types such as microglia playing a critical role in the pathogenesis of PD^57,58^. In particular, chronic microglial activation is suggested as key pathophysiological feature of many neurodegenerative disorders including PD^59^. Following a previously described protocol^60^, we differentiated the subclones of WIBR3 (WIBR3-S1, WIBR3-S2, WIBR3-S3) into differentiated microglia-like cells (iMGs). In this protocol, hPSCs are initially induced to myeloid intermediates and subsequently differentiated to microglia through the addition of cytokines normally secreted from neurons and astrocytes including IL-34, M-CSF, and TGF-β1 (Figure 2F). All WIBR3 sub-clones robustly generated microglial precursors (iMPs) at a high purity, evidenced by the presence of 91.3% CD11b/CD45 and 79.6% CD14/CD16 positive cells (Figure 2G–J). Moreover, terminal differentiation yielded cells expressing key markers for mature microglia such as IBA1, CX3CR1, and P2RY12, as confirmed through immunostaining (Figure 2K–N, Supplemental Figure 5). Collectively, these findings underscore the suitability of WIBR3 hESCs as a model to study the contribution of different cell types to PD pathology.

### Genetic engineering of PD-associated mutations

**Figure 3.**
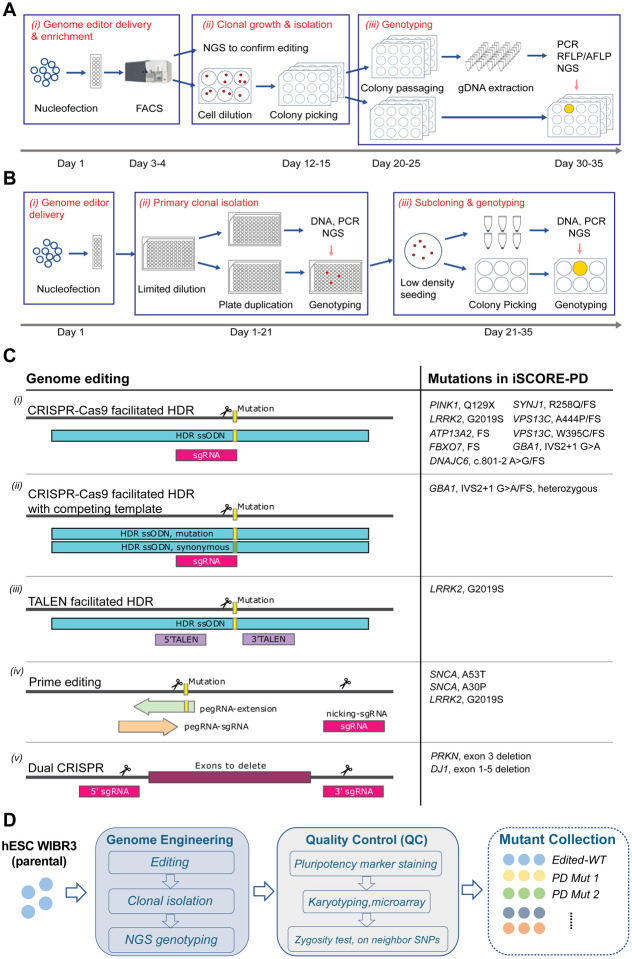
Gene editing workflow to generate iSCORE-PD Collection. (A) Schematic illustrating genome editing pipeline A. This approach involves *(i)* FACS-based enrichment of nucleofected cells containing the gene editing reagents including a fluorescent reporter, *(ii)* the isolation of clonally expanded cell lines and *(iii)* the NGS-based genotyping to identify correctly edited cell lines. (B) Schematic illustrating genome editing pipeline B. This approach utilizes a high-throughput cell isolation system. This approach includes *(i)* nucleofection of the gene editing reagents, *(ii)* the plating of cells in a limited dilution (~10 cells/well) to isolate wells containing correctly targeted cells by NGS and *(iii)* subcloning, expansion and NGS-based genotyping to isolate correctly targeted clonal cell line. (C) Table summarizing the gene editing strategies used to generate the iSCORE-PD collection. These include: *(i)* CRISPR/Cas9 facilitated homology directed repair (HDR) using ssODNs containing the desired genetic modification as repair template for CRISPR/Cas9 induced double strand break. *(ii)* The use of competing HDR templates (ssODNs) containing synonymous mutations in the gRNA-target site to favor the generation of heterozygous over homozygous mutations. *(iii)* TALEN-facilitated HDR using ssODNs containing the desired genetic modification as repair template for CRISPR/Cas9 induced double strand break. *(iv)* Prime editing approach to insert the PD-associated point mutations into hESCs. *(v)* Dual CRISPR approach using 3’ and 5’ sgRNAs flanking the desired deletion to recreated large genomic structural alterations identified in PD patients. (D) Overview depicting genome engineering and quality control steps in the generation of the iSCORE-PD collection.

The goal of this work is to establish a comprehensive collection of cell lines carrying mutations associated with PD within a well-defined genetic background. This requires precise and robust gene editing approaches to insert the desired genetic alterations in hPSCs. We previously demonstrated that WIBR3 cells can be efficiently genetically modified using either CRISPR/Cas9, TALEN or prime editing-based genome engineering approaches^35,61,62^. CRISPR/Cas9-based editing is effective for introducing targeted genomic deletions and biallelic alterations, while prime editing is highly efficient in introducing heterozygous modifications, required for modeling dominantly inherited disease-associated alleles^61^. To create cell lines carrying PD-associated genetic alterations in WIBR3 hESCs we employed two different editing pipelines: Pipeline A shown in Figure 3A utilizes FACS-enrichment post-nucleofection to purify effectively transfected cells, followed by clonal expansion and genotyping to allow the isolation of clonal, correctly targeted lines (estimated time for the editing pipeline is 30–35 days).; Pipeline B shown in Figure 3B uses nucleofection, limited dilution, and next generation sequencing (NGS)-based genotyping to identify desirable edits in a 96-well plate system. This integrated workflow allows the efficient isolation and purification of correctly edited clonal cell lines, even at low frequency, within a shorter time frame compared to previous approaches (21–35 days).

Given the identification of several disease-causing alleles within most PD-linked genes, the selection and prioritization of specific alleles for gene editing within each gene was based on the confidence of pathogenicity for each mutation, the allele prevalence, and the feasibility of the editing strategy. As described in detail below for each gene, we employed three general editing strategies to closely recreate the genomic alterations identified to be causal or high-risk factors for PD. These editing strategies include *(i)* the precise insertion of point mutations using CRISPR/Cas9, TALEN or prime editing approaches to recreate specific PD-associated missense mutations (heterozygous and/or homozygous), *(ii)* the insertion of small indels to create frameshift (FS) or premature stop mutations using CRISPR/Cas9, and *(iii)* dual guided RNA (gRNA)-mediated CRISPR/Cas9 deletions to create genomic deletions identified in PD patients (Figure 3C).

### Quality control (QC) of genetically modified hPSCs

One significant challenge associated with any genome editing approach is the risk of introducing unintended on- and off-target genetic modifications in the edited cell lines. Additionally, it is well-established that clonal expansion and *in vitro* culture of hPSCs can lead to the acquisition of genetic alterations that provide growth advantages^51–53,63–66^. Consequently, there is a consensus in the field that all gene-edited hPSC-derived disease models should undergo a rigorous quality control process to validate the pluripotent properties of the edited cell lines and ensure the absence of major gene editing- or culture-induced genetic alterations. As part of this collection, all genome-edited cell lines underwent a comprehensive quality control process, as outlined in Figure 3D. Following CRISPR/Cas9 or prime editing-based genome editing and subsequent clonal expansion, individual correctly targeted clones were initially identified using either Sanger sequencing or NGS. Subsequently, all correctly targeted clonal lines were expanded, cryopreserved at a low passage number post-genome editing, and subjected to testing for the expression of pluripotency markers OCT4, SSEA4, and alkaline phosphatase by immunocytochemistry. To confirm a normal karyotype and assess the overall genomic integrity, genome-edited clonal hESC lines underwent standard aCGH genotyping and were analyzed using a modified high density Illumina Infinium Global Diversity Array (GDA) Neuro booster Array (NBA). This analysis aimed to exclude cell lines with large genome editing-induced structural alterations or complete chromosomal loss compared to the genome of the parental WIBR3 cell line (complete high-density array genotyping data is available at https://www.amp-pd.org/).

A frequently overlooked challenge associated with genotyping approaches based on PCR amplification of the target locus is the common failure to detect loss of heterozygosity (LOH)^67^. LOH can result from large deletions or the loss of entire chromosome fragments distal to the targeting site induced by the genome editing process. In order to rule out LOH, in cell lines that appear to be homozygously edited based on the detection of a single alleles by NGS, we introduced an additional quality control step, using either a southern blot or SNP-PCR based analysis, to validate the presence of two alleles at the targeting site as described in detail below for each gene. Using this analysis, we identified LOH in 4 out of 13 tested clonal cell lines classified initially as correctly edited with two identical alleles at the target site. This data underscores that LOH is a significant complication arising from genome editing, emphasizing the importance of incorporating LOH testing as a critical component of the quality control process for genome-edited hPSC lines.

A summarized list of the genes, mutations, and number of cell lines in the iSCORE-PD collection can be found in Table 1. For detailed information on each cell line in the iSCORE-PD collection, see Supplemental Table 3 and Supplemental Figures 6–17 (pipeline used, gene and mutation targeted, genome engineering technique, genetic description, expected protein products and quality control results). Overall, 25.8% (17/66) of isolated clonal cell lines with correct NGS-confirmed genotype were excluded from our collection. As summarized in Supplemental Table 4, the reasons include chromosomal and structural alterations (18.2% - 12 out of 66), lack of expression of a pluripotency marker (1.5% - 1 out of 66) and LOH (6.1% - 4 out of 66). It is important to note that the frequency of chromosomal and large structural abnormalities was higher in clones generated by double strand break-based genome editing (CRISPR and TALEN, 25.0% - 11 out of 44) compared to prime editing (5.3% - 1 out of 19). Similarly, deletions resulting in LOH at the targeted locus were frequent in double strand break-based genome edited cell lines (36%, 4 out of 11 tested) but absent in prime edited cell lines. Given that any genetic alteration induced by cell culture or genome editing can impact the biological properties of hPSCs and disease phenotypes, this data emphasizes the necessity for a comprehensive quality control in genome engineering. Considering that clonally derived cell lines are susceptible to cell culture-induced genetic drift and acquire additional genetic and epigenetic alterations over time, we advocate using multiple independently gene-edited clonal lines for each genotype to account for this variability. In addition, we recommend performing routine quality control analysis to detect long term culture induced genetic aberrations. This approach ensures a robust assessment of disease-relevant phenotypes *in vitro*, acknowledging the potential variability that may arise during prolonged cell culture and genome editing processes.

### iSCORE-PD collection of isogeneic hPSC lines carrying PD-associated mutations.

For our initial iSCORE-PD collection, we prioritized engineering cell lines carrying mutations in high confidence PD genes^4^. The specific modifications for each gene were selected based on information in the MDSgene database^68^ (https://www.mdsgene.org) and the currently available literature as outlined for each gene below. Overall, 55 clonal cell lines carrying PD-associated mutations in 11 genes linked to PD along with isogenic control lines passed all the above-described quality control steps and have become part of the iSCORE-PD collection (Table 1, Supplemental Table 3). All cell lines will be available to the scientific community through the WiCell Research Institute (https://www.wicell.org/). Our intention is to continue to expand this collection in the future. At the time of publication, iSCORE-PD includes the following cell lines:

#### Control cell lines

Together with the parental WIBR3 cell line, we provide a set of sub-clones with this collection (WIBR3-S1, S2, S3) (Figure 1, Supplemental figure 1). In addition, we included WIBR3 cell lines that were isolated as part of the standard genome editing pipeline but did not exhibit any genetic modifications at the targeted locus. We consider these as “edited wild-type” cells (EWT), which are the best experimental control to account for any non-specific changes caused by the gene editing process (EWT1–3: prime editing controls - Pipeline B, EWT4–5: CRISPR/Cas9 controls - Pipeline B and EWT6–8: prime editing controls - Pipeline A) (Table1, Supplemental Table 3, Supplemental Figure 6).

#### SNCA (PARK1)

The *SNCA* gene encodes the alpha-synuclein (ɑ-Syn) protein. The discovery that mutations and copy number increases of the *SNCA* gene linked to familial forms of PD, along with the identification of non-coding variants in the *SNCA* locus as a risk factor for sporadic PD, indicate a central pathogenic role for this protein^69^. Moreover, fibrillar ɑ-Syn is the major component of Lewy bodies and changes in the dosage and aggregation and clearance properties of ɑ-Syn are thought to be central pathogenic drivers of PD^2–4,43,69^. We established prime editing reagents to efficiently introduce the A30P or A53T mutation in the *SNCA* gene, which are both linked to autosomal dominant forms of PD^61,70,71^. Using this approach (Supplemental Figure 7A,D), we generated additional cell lines so that the iSCORE-PD collection now includes 3 cell lines carrying the A53T mutation (heterozygous) (Supplemental Figure 7B,C) and 4 cell lines carrying the A30P mutation (3 heterozygous and 1 homozygous) (Supplemental Figure 7E,F). All cell lines passed the above-described quality control steps. In addition, we performed SNP-PCR followed by Sanger sequencing based zygosity analysis to exclude LOH in the homozygous *SNCA* A30P line (Supplemental Figure 6G).

#### *PRKN* (PARK2)

Mutations in the *PRKN* gene, which encodes the E3 ubiquitin-protein ligase parkin (Parkin), are the most frequent cause for autosomal recessive PD^72^. The first genetic alterations in *PRKN* linked to PD were distinct large-scale deletions^73^. Since then, a wide range of deletions and point mutations have been identified^74^. Molecular alterations in Parkin impact a wide range of molecular and cellular functions including responses to oxidative stress, mitochondrial membrane potential, mitochondrial motility, contact sites with the endoplasmic reticulum and the regulation of inflammatory responses^75,76^. Homozygous and compound heterozygous deletions of Exon3 (Ex3del) are repeatedly found in families with PD, and Ex3del is now a confirmed pathogenic variant of *PRKN*^74,77^. We used a dual CRISPR/Cas9 approach (Figure 3C[v]) targeting each side of Exon3 to generate 3 cell lines that are homozygous for the Ex3del (Supplemental Figure 8A–C). Subsequent southern blot analysis to exclude LOH revealed that one cell line (WIBR3_PRKN_X3DEL_B1–3) carries a larger Ex3del (~3.8 kb, Supplemental Figure 8D,E), which we fully characterized by NGS sequencing (Supplemental Figure 8F). We included this cell line in the iSCORE-PD collection since the flanking exons 2 and 4 are not affected by the deletion and thus, no significant consequences are expected of the extended deletion. No heterozygous clones have been included in this collection since these genotypes are frequent in control populations and not associated with a higher risk of PD^78^.

#### *PINK1* (PARK6)

Mutations in *PINK1* were first associated with PD in 2004 following earlier studies linking the PARK6 genomic region with increased risk for PD^79^. Subsequently, a large number of point mutations, frameshift mutations and deletions have been identified, predominantly affecting the activity of the kinase domain^74^. This indicates that *PINK1* loss-of-function is the cause for early-onset, autosomal recessive PD. Serine/threonine-protein kinase PINK1 plays a crucial role in mitochondrial quality control by regulating mitochondrial homeostasis and clearance^80^. Based on the segregation of the Q129X and a related Q129fsX157 mutation in *PINK1* observed in two large families with PD^81^, we generated 3 cell lines that are homozygous for the Q129X mutation in *PINK1* (Supplemental Figure 9A–C). To exclude LOH, we performed SNP-PCR followed by Sanger sequencing based zygosity analysis (Supplemental Figure 9D). Heterozygous *PINK1* mutant cell lines were not included in the iSCORE-PD collection, as the heterozygous genotype is not considered a risk factor for PD.

#### *DJ1* (PARK7)

Since its initial association with PD^82^, multiple point mutations and genomic rearrangements in *PARK7* (*DJ1*), encoding the Parkinson disease protein 7, have been identified as a rare cause for autosomal recessive PD^74^. While the exact function of this enzyme remains largely unknown, Parkinson disease protein 7 is implicated in regulating transcription, cell growth and oxidative stress response pathways linked to cell survival and apoptosis^83–85^. Based on the identification of homozygous deletions of either exon 5^86^ or exon 1 to 5^87^ in families with PD, we used a dual CRISPR/Cas9 approach to closely recapitulate the Exon 1 to 5 deletion (Supplemental Figure 10A–C). Southern blot analysis was performed to exclude LOH in the homozygous exon 1–5 deleted cell lines (Supplemental Figure 10D,E).

#### *LRRK2* (PARK8)

Mutations in the *LRRK2* gene, encoding the leucine rich repeat serine/threonine-protein kinase 2, were first linked to PD in 2004^88^. Over the years, more than 100 different variants of the gene have been described^89^, establishing *LRRK2* coding variants as the most frequently mutated gene linked to dominant and sporadic forms of PD. Among these mutations, G2019S is the most common substitution identified across populations^90^, found in approximately 4% of dominantly inherited familial PD cases, in both heterozygous and homozygous forms, and around 1% of sporadic PD cases^91^. This variant increases the kinase activity of leucine rich repeat serine/threonine-protein kinase 2, affecting a wide range of cellular and molecular processes including vesicular trafficking and cytoskeleton dynamics, autophagy and lysosomal degradation, neurotransmission, mitochondrial function, and immune and microglial responses^92,93^. We recently established CRISPR/Cas9, TALEN and prime editing reagents to efficiently introduce the G2019S mutation in the *LRRK2* gene^61^. Using this approach (Supplemental Figure 11A), we generated additional cell lines so that the iSCORE-PD collection now includes 5 clones carrying G2019S (4 heterozygous and 1 homozygous) (Supplemental Figure 11B–C). We performed SNP-PCR followed by Sanger sequencing based zygosity analysis to exclude LOH in the homozygous *LRRK2* G2019S line (Supplemental Figure 11D).

#### *ATP13A2* (PARK9)

Mutations in the *ATP13A2* gene, encoding the polyamine-transporting ATPase 13A2 (ATP13A2) protein, were identified as a cause for PD in the PARK9 locus in 2006, with this genomic region previously associated with Kufor Rakeb disease^94^. Various mutations in the gene result in a truncated ATP13A2 protein causing it miss-localization from the lysosome to the endoplasmic reticulum, where it accumulates before being eventually targeted for proteasomal degradation in the cytpplasm^94,95^. Loss of ATP13A2 is linked to mitochondrial and lysosomal dysfunction, as well as to the accumulation of ɑ-Syn due to the dysregulation of proteasomal and autophagy-mediated protein degradation^96–99^. Based on the identification of a homozygous frameshift mutation (T367RfsX29) in a family with recessive PD^100^, we used CRISPR/Cas9-based genome editing to engineer the frameshift T367Rfs mutation in the *ATP13A2* gene (Supplemental Figure 12A). Given the recessive inheritance pattern of the gene, our collection contains 5 clones with the T367Rfs frameshift mutation, predicted to result in a truncated loss of function protein similar to that observed in PD patients (Supplemental Figure 12B,C). We performed SNP-PCR followed by NGS sequencing-based zygosity analysis to exclude LOH in the homozygous edited cell lines (Supplemental Figure 12D).

#### *FBXO7* (PARK15)

Mutations in the *FBXO7* gene, encoding the F-box only protein 7 (FBOX7), were first linked to autosomal recessive PD in 2008^101^. The most notable homozygous *FBXO7* variant, found in multiple family pedigrees with PD, is the truncating R498X premature stop mutation located in the proline-rich region of the FBOX7 protein^102^. This mutation disrupts the interaction of FBOX7 with PINK1 and Parkin^103–105^ and results in abnormal localization and reduced stability of the truncated FBXO7 protein. This causes disruption of mitophagy and leads to mitochondrial aggregation^103,106,107^. We used CRISPR/Cas9-based genome editing to engineer a frameshift mutation which leads to a premature stop and is predicted to result in truncated FBXO7, similar to the protein in patients carrying the R498X mutation (Supplemental Figure 13A–C). We performed southern blot and SNP-PCR followed by NGS sequencing based zygosity analysis to exclude LOH in the homozygous edited cell line (Supplemental Figure 13D–F).

#### *DNAJC6* (PARK19)

The *DNAJC6* gene encodes for the protein putative tyrosine-protein phosphatase auxilin (auxilin), which belongs to the DNAJ/HSP40 family of proteins, which regulate molecular chaperone activity. *DNAJC6* was initially linked to very early onset, autosomal recessive PD in 2012^108^ through the identification of a mutation that affects splicing and the expression of auxilin. Consistent with a role as co-chaperone to recruit HSC70 to clathrin-coated vesicles, hESC models show that *DNAJC6* alterations result in loss of auxilin protein, leading to the accumulation of clathrin, reduced vesicular transport, and the degeneration of midbrain dopaminergic neurons^109^. Furthermore, these alterations are associated with ɑ-Syn aggregation, mitochondrial and lysosomal dysfunction, and lipid defects^109–111^. We used a CRISPR/Cas9-based approach to recapitulate the effect of the c.801 −2A>G splice acceptor site mutation by either precise insertion of this mutation or a frameshift modification (Supplemental Figure 14). Similar frameshift modifications were previously shown to recapitulate the loss of *DNAJC6* expression in hESC-derived neuronal cells^109^, similar to what is observed in *DNAJC6* variant carriers^108^.

#### *SYNJ1* (PARK20)

*SYNJ1* encodes Synaptojanin-1 and was first linked to early onset autosomal recessive PD in 2013^112,113^. Synaptojanin-1 is predominantly expressed in neurons and is concentrated in presynaptic terminals. Homozygous *SYNJ1* mutations are linked to alterations in lipid metabolism and vesicle trafficking^112,113^, as well as defects in autophagosome maturation^114^. We used a CRISPR/Cas9 approach to insert the R258Q substitution, which was identified in a homozygous state in several independent families with PD^112,113,115^ (Supplemental Figure 15A). The iSCORE-PD collection includes clones that are homozygous for the R258Q mutation in *SYNJ1* or compound heterozygous for the R258Q and a frameshift allele at the same location. Both genotypes are expected to recapitulate the modification in *SYNJ1* associated with disease (Supplemental Figure 15B,C). We performed southern blot based zygosity analysis to exclude LOH in the homozygous edited cell line (Supplemental Figure 15D–F).

#### *VPS13C* (PARK23)

Mutations in *VPS13C* were initially identified as the cause of autosomal recessive early-onset PD in 2016^116^. The initial functional analysis revealed that disruption of Intermembrane lipid transfer protein VPS13C (VPS13C) causes decreased mitochondrial membrane potential, mitochondrial fragmentation, increased respiration rates, exacerbated PINK1/Parkin-dependent mitophagy, and transcriptional upregulation of *PRKN* in response to mitochondrial damage^116^. As the VPS13C protein is critical for the transport of lipids between the ER and endosome/lysosome, as well as for lipid droplet formation^117,118^, loss of VPS13C causes the accumulation of lysosomes and altered lipid profiles^119^. We used CRISPR/Cas9-based editing to introduce the W395C and A444P variants into the *VPS13C* gene (Supplemental Figure 16A,E), both of which are found as homozygous or compound heterozygous mutations in PD patients^120,121^. While *VPS13C* is thought to cause PD through a loss of function mechanism, we included additional frameshift alleles in the iSCORE-PD collection to allow further investigation of the loss of function mechanism (Supplemental Figure 16B,C,F,G). We performed SNP-PCR followed by Sanger sequencing based zygosity analysis to exclude LOH in the homozygous *VPS13C* A444P lines (Supplemental Figure 16D).

#### GBA1

The *GBA1* gene codes for the enzyme Lysosomal acid glucosylceramidase, which is essential for maintaining glycosphingolipid homeostasis. While homozygous or compound heterozygous pathogenic variants in *GBA1*, associated with reduced glucosylceramidase activity, cause autosomal recessive Gaucher disease, heterozygous carriers of pathogenic *GBA1* variants have a high risk of developing PD^122,123^. *GBA1* mutations are currently considered the strongest risk factor for PD and Lewy body dementia with odd ratios between 1.4 to >10^124^. Given that *GBA1* mutations are present in approximately 3–20% of sporadic PD patients across different populations, *GBA1* represents the most prevalent genetic risk factor for PD^124^. Over 300 mutations in *GBA1* with variable risks for developing PD have been reported^123^. Among them, the splice site IVS2+1 mutation, causing missplicing and loss of *GBA1* expression, represents one of the most pathogenic alleles for PD^122,123^. To insert the IVS2+1 in the *GBA1* gene, we devised a CRISPR/Cas9-based targeting strategy that allows specific targeting of the *GBA1* gene and not the nearby highly homologous *GBAP1* pseudogene (Supplemental Figure 17A). To identify correctly targeted clones, we used a genotyping strategy that can conclusively distinguish between the *GBA1* and *GBAP1* pseudogene based on small sequence variation (Supplemental Figure 17B–D). Using this approach, we generated 2 heterozygous and 2 homozygous edited cell lines carrying the IVS2+1 mutation in *GBA1*. In addition, to allow comparison between the IVS2+1 and a loss of function allele, we also included cell lines with a frameshift mutation at the same genomic location in the iSCORE-PD collection.

## Discussion

Population genetics and advancements in sequencing technologies have significantly contributed to our understanding of the genetic architecture of complex diseases and have identified numerous genetic variants linked to the development and progression of diseases such as PD. While each of these variants could provide valuable insights into the pathophysiology of the underlying disease, unraveling their function within a genetically heterogeneous population remains challenging due to additional effects from undefined genetic modifier loci. To overcome this limitation, we present an experimentally tractable human cellular model system that facilitates molecular and cellular studies in a genetically controlled platform. For this, we have generated a collection of isogenic hESC lines that carry monogenic or high-risk PD-associated mutations. Similar to the development of inbred animal models, which have proven instrumental in establishing robust genotype-phenotype correlations and enabled the comparison of such phenotypes across research groups, our isogenic cell line collection offers the opportunity to directly compare the phenotypic effect of PD-associated mutations in a genetically controlled system across genes and laboratories.

The establishment of an isogenic cell line collections involves two pivotal components, both crucial for the effective implementation of this approach; *(i)* a thorough characterization of the parental hPSC line and *(ii)* the establishment of a genome editing platform enabling the efficient engineering of genetic alterations similar to those found in patients. Regarding the hPSC line, we have conducted a comprehensive analysis of the parental WIBR3 hESC line and demonstrated that the genomic integrity of this cell line can be sustained over extended periods in culture. We also show that WIBR3 cells are amenable to multiple rounds of clonal expansion and genome engineering. In addition, high-density genotyping and long-read WGS show that the WIBR3 cell line does not carry major structural or genetic alterations impacting genes with known relevance to PD. Importantly, we demonstrate that WIBR3 cells can efficiently generate PD-relevant cell types *in vitro* using established differentiation protocols. Thus, WIBR3 cells are a highly characterized female reference hESC line that complements current hPSC lines for modeling neurodegenerative diseases.

To generate this collection, we established CRISPR/Cas9, TALEN and prime editing pipelines in hPSCs, enabling the highly efficient and multiplexed introduction of a broad range of genetic alterations known to be associated with a given disease, ranging from heterozygous and homozygous single nucleotide variants to large structural genomic deletions. During the process of establishing this collection, we made several key observations. Notably, we recognized that both CRISPR/Cas9 and prime editing approaches necessitate a comprehensive quality control (QC) process beyond the validation of the intended genomic modification. Consequently, all the described hESC lines underwent a rigorous quality control procedure, which included the validation of pluripotency using immunohistochemistry, the exclusion of karyotypic and structural aberrations using standard aCGH arrays, high-density genotyping arrays and zygosity analysis at the targeted genomic locus. The need for this quality control is evident from the high number of genome edited cell lines that were excluded due to structural aberrations and loss of heterozygosity (LOH) at the target locus (Supplemental Table 4).

A key finding across the derivation of all cell lines was that the frequency of karyotypic and structural aberrations as well as LOH of the edited locus was substantially more frequent in CRISPR/Cas9 than in prime edited cell lines. In fact, LOH was exclusively seen in CRISPR/Cas9-edited clonal hESCs. While this is the first time that this has been formally reported across a large cohort of gene edits, the finding might not be unexpected. In contrast to CRISPR/Cas9-based genome editing, prime editing does not require a double strand break (DSB) at the target site to insert genetic modifications^125^. Instead, prime editing directly repairs a prime editor-induced single strand DNA break^125^. These results confirm our previous observations, that prime editing has substantial advantages over CRISPR/Cas9-based approaches for introducing point mutations and small structural modifications in hPSCs^61^. Furthermore, we strongly recommend including a zygosity analysis, specifically to exclude LOH at the target locus, as a critical step in the quality control pipeline of genome engineered hPSCs.

While the cell lines described here currently focus on coding risk variants with large effect size linked to monogenic PD^4^, we envision that we and other researchers can expand this collection to eventually incorporate GWAS-identified risk variants with lower effect size. Such an expansion could provide functional insights into how these primarily non-coding sequence variants affect similar cellular and molecular pathways as implicated in monogenic PD. To facilitate such efforts, all generated cell lines will be made available with the support of the Aligning Science Across Parkinson’s (ASAP) initiative and the Michael J. Fox Foundation through the WiCell Research Institute. We anticipate that the subsequent biological analysis of this comprehensive collection and its future expanded forms, involving numerous research groups with diverse expertise, can provide a unified understanding of how genetic risk variants functionally contribute to the pathogenesis of PD. We predict that this collaborative effort has the potential to accelerate the development of novel therapeutic strategies for PD.

## Limitations of this study

Each genome is inherently unique, carrying a distinct combination of sequence variants and genetic alterations that can influence the development and pathology of complex diseases such as PD. Consequently, there is no single cellular model that can fully recapitulate all the molecular and cellular features of such disorders. Given this limitation, it will become necessary to expand the described approach to include additional cell lines with diverse genetic backgrounds to fully dissect the pathobiology of PD. Moreover, it is widely recognized that genetic engineering and *in vitro* culture of cell lines lead to the acquisition of genetic and epigenetic modifications, potentially affecting the phenotypical analysis of *in vitro*-derived cell types, irrespective of the disease genotype. While using multiple independently edited clonal cell lines alongside continuous quality control measures can mitigate many such random alterations, we cannot entirely exclude remaining systematic genetic and epigenetic off-target modifications associated with specific gene editing approaches.

## Methods.

### hPSCs culture

hESCs were maintained on irradiated or mitomycin C-inactivated mouse embryonic fibroblast (MEF) monolayers as described previously^61^ with daily changes of hESC media (Dulbecco’s Modified Eagle Medium/Nutrient Mixture F-12 (DMEM/F12; Thermo Fisher Scientific) supplemented with 15% fetal bovine serum (Hyclone), 5% KnockOut Serum Replacement (Thermo Fisher Scientific), 1 mM glutamine (Invitrogen), 1% nonessential amino acids (Thermo Fisher Scientific), 0.1 mM β-mercaptoethanol (Sigma) and 4 ng/ml fibroblast growth factor (FGF) (Thermo Fisher Scientific/Peprotech), 1×Penicillin-Streptomycin (Thermo Fisher Scientific). All hESCs cultures were maintained in a cell culture incubator under low oxygen conditions (95% CO2, 5% O2). Cultures were passaged as aggregates every 5–7 days using a collagenase IV solution (Gibco) to detach hESC colonies. All cell lines are tested routinely for mycoplasma. Detailed protocols for culturing of hESCs on MEF feeders can be found on protocols.io (https://doi.org/10.17504/protocols.io.b4pbqvin ; https://doi.org/10.17504/protocols.io.b4msqu6e). All hESCs cultures were adapted to feeder-free culture conditions before starting in vitro differentiation experiments. hESCs were maintained on geltrex/matrigel coated plates in mTeSR plus medium (Stem Cell Technologies) in a cell culture incubator under low oxygen conditions (95% CO2, 5% O2) as described previously^61^. Cells were passaged regularly as aggregates either manually or using ReLeSR (Stem Cell Technologies) to detach hESC colonies. Detailed protocols for feeder-free culturing of hPSCs can be found on protocols.io (https://doi.org/10.17504/protocols.io.b4mcqu2w).

### Collecting cell pellets for DNA and RNA extraction

hESCs colonies cultured on MEFs were harvested by collagenase IV and washed twice through an 80 μm cell strainer to further remove MEFs. Collected colonies were pelleted by centrifugation and snap frozen in liquid nitrogen.

### Array genotyping and data processing

Genomic DNA was isolated from cell pellets using the DNeasy Blood & Tissue Kit (QIAGEN; 69504). Genotyping was performed using the Neuro Booster Array (NBA) with best practices guidelines for the Infinium Global Diversity Array^126^. Genotyping data was processed using GenomeStudio and subsequent genotype calls, B-allele frequency and LogR ratio values were used for genomic integrity assessments. When cell lines carrying a genomic edit were present on the NBA, genotype calls were compared to confirm the edit. Genome-wide genotyping calls were compared with the PacBio HiFi WGS variants to assess large genomic events across the two data types using PLINK (v1.9)^127^. The B-allele frequency and LogR ratio values were processed and plotted using the GWASTools package in R (v3.6.1)^128^.

### Long-read sequencing and data processing

#### Oxford Nanopore Technologies DNA extraction, library preparation, and sequencing

Ultra-high molecular weight DNA (UHMW) was extracted from the WIBR3 (parental) hESC line (5 × 10^6^ cells) following the Circulomics/Pacific Biosciences (PacBio) UHMW DNA Nanobind Extraction protocol (Circulomics/PacBio, no longer available) with the Nanobind CBB Kit (PacBio, SKU 102-301-900) and the UHMW DNA Aux Kit (Circulomics/PacBio, NB-900-101-01, no longer available). The extracted DNA was checked using the Qubit dsDNA BR assay (Invitrogen, Q32850) to ensure proper extraction occurred. The extracted UHMW DNA was then taken straight into library preparation for sequencing using Oxford Nanopore Technologies (ONT) SQK-ULK001 Kit and the Nanobind Ultra Long Library Preparation Kit (Circulomics/PacBio, NB-900-601-01, no longer available). The library was split into 3 tubes of 75 μl each, and each tube loaded on a flow cell. After 24 hours, 75 μl of the sequencing library was pulled out of each flow cell and reloaded on a fresh flow cell. This process was repeated one more time for a total of 9 separate R9.4.1 PromethION flow cells.

#### Pacific Biosciences DNA extraction, library preparation, and sequencing

High molecular weight (HMW) was extracted using PacBio’s Nanobind CBB Kit (Pacbio, 102-301-900) from 2 × 10^6^ cells with the Nanobind adherent cultured cells protocol. After extraction, DNA concentration was quantified using the Qubit dsDNA BR assay (Invitrogen, Q32850), sized with a Femto Pulse System (Agilent, M5330AA), and size selected with the PacBio SRE Kit (Pacbio, SKU 102-208-300). Following quality control, the extracted DNA was sheared to a target size of 18–20 kb using the Megaruptor 3 (Diagenode, B060100003). After confirmation of correct sizing, the library preparation was performed SMRTbell prep kit 3.0 (PacBio, 102-141-700) with a PEG wash. The library was sequenced on a Revio flow cell with a 24 h movie time.

#### Long read sequencing Data Analysis

ONT sequencing runs were basecalled on NIH’s HPC (Biowulf) using Oxford Nanopore’s Guppy (v6.1.2) in super accuracy mode with the *dna_r9.4.1_450bps_modbases_5mc_cg_sup_prom.cfg* configuration file and the –bam_out option to preserve methylation tags. The basecalled bams were then converted to fastqs using Samtools (v1.17)^129^ (samtools fastq -TMm, Ml) and mapped to hg38 using Minimap2 (v2.24)^130^ with ONT flags. Data from all flow cells was merged after mapping using samtools (v1.17). Then, we used PEPPER-Margin-DeepVariant (v.0.8)^131^ to call small variants (<50bp) and phase our variant calls and alignments. We then used our phased alignment, to produce haplotype-specific methylation calls using Modbamtools (v0.4.8)^132^ and Nanopore’s modbam2bed (GitHub - epi2me-labs/modbam2bed). Lastly, structural variants (SVs) were called using Sniffles2 (v2.2)^133^ with default settings. PacBio Revio HiFi data was processed according to general best practices. Data was mapped using Minimap2 (v2.24) using PacBio flags. Small variant calls generated by Clair3 (v1.0.4) (GitHub - HKU-BAL/Clair3: Clair3 - Sym...) with PacBio flags and SV calls were generated by Sniffles2 (v2.2)^133^. Small variants were filtered for DP>15 and GQ>20 using bcftools (v1.17)^129^ and annotated with ANNOVAR (v.2022-06-08)^134^ to assess the presence of potential pathogenic variants. In addition Alzheimer’s disease^135^ and Parkinson’s disease genetic^43^ risk scores (excluding UK Biobank summary statistics) were calculated to assess the cumulative risk score using plink (v2.0) for disease and compared with participants from the UK Biobank diagnosed with AD and PD^136^. Only SV calls labeled as “PASS” were kept for both ONT and PacBio data. The “PASS” SV calls were then annotated with ANNOVAR (v.2022-06-08)^134^ and coding variants were subset. Then, we used Truvari (v4.4.0) (GitHub - ACEnglish/truvari: Structura...) to merge structural variant calls between the ONT and PacBio datasets both for all variants as well as only coding variants. Numbers on variant type distribution were generated using SURVIVOR (v1.0.7) (GitHub - fritzsedlazeck/SURVIVOR: Too...). Lastly, the SV overlaps were further annotated using SVAnna (v1.0.4) (GitHub - TheJacksonLaboratory/SvAnna:...) with the phenotype terms HP:0002180 (neurodegeneration) and HP:0000707 (abnormality of the nervous system). SVs of interest were plotted using samplot (v1.3.0) (GitHub - ryanlayer/samplot: Plot stru...).

### Molecular cloning

Molecular cloning was carried out as described previously^61^ following standard cloning protocols^137^. As described^125^, pegRNA plasmids for prime editing were cloned by ligating annealed oligonucleotide pairs (Supplemental table 5) into the BsaI-digested pU6-peg-GG-acceptor (pU6-pegRNA-GG-acceptor was a gift from David Liu. Addgene #132777; http://n2t.net/addgene: 132777; RRID:Addgene_132777). Prime editing nicking guide plasmids (ngRNAs) were cloned by ligating annealed oligonucleotide pairs (Supplemental table 5) into the BsmBI-digested pBPK1520 plasmid (BPK1520 was a gift from Keith Joung. Addgene#65777; http://n2t.net/addgene: 65777; RRID:Addgene_65777)^138^. For CRISPR/Cas9 based genome editing, the Cas9 expressing gRNA plasmids were cloned by ligating annealed oligonucleotide pairs (Supplemental Table 5) into the BbsI-digested px330-GFP or px330-mCherry as described previously^16^. For TALEN mediated genome editing, we used previously described heterodimeric TALEN pairs to insert the G2019S into the LRRK2 gene^40^. Sequence information for all oligonucleotides (Integrated DNA Technologies, IDT) used to generate plasmids can be found in Supplemental Table 5.

### Genome editing of hESCs

As outlined in Figure 3C, genome editing of WIBR3 hESCs was performed using either plasmid or ribonucleoprotein particle (RNP) based CRISPR/Cas9 or prime editing approaches as described previously^61^ using the following procedures:

#### Nucleofection

hESCs cultured on MEFs were pre-treated with 10 μM ROCK inhibitor (Y27632, ToCris) 1-day before nucleofection (2–3 hours at a minimum is recommended). Cells were collected by collagenase IV (Thermo Fisher Scientific) followed by Accutase (Thermo Fisher Scientific) to dissociate hESCs into a single cell solution. 5 × 10^5^ to 1 × 10^6^ cells were resuspended in 20μL of nucleofection solution (P3 Primary Cell 4D-Nucleofector^™^; Lonza) and nucleofected (Lonza 4D nucleofector TM Core + X Unit, program CA-137) using the following genome editing reagents for the corresponding edits described in Figure 3: *(1)* Plasmid based CRISPR-Cas9 facilitated HDR: 200 ng gRNA plasmids (px330-GFP), 700 ng ssODN. *(2)* Plasmid based dual CRISPR: 500 ng 3’-gRNA plasmid (px330-GFP) and 500 ng 5’-gRNA plasmid (px330-mCherry). *(3)* TALEN facilitated HDR: 100 ng LRRK2-TALEN-TA01L and 100 ng LRRK2-TALEN-TA03R, 700 ng ssODN, 100 ng pEGFP-N1 (Clontech). *(4)* Plasmid based prime editing: 500 ng pCMV-PE2-GFP (a gift from David Liu, Addgene#132776)^115^, 330 ng pU6-pegRNA and 170 ng pBPK1520-ngRNA. *(5)* RNP-based CRISPR-Cas9 facilitated HDR: 80 pmol purified Cas9 protein (QB3 Macrolab, UC Berkely), 300 pmol chemically modified synthetic sgRNA (Synthego) and 100 pmol ssODN HDR template. *(6)* RNP-based dual CRISPR: 80 pmol purified Cas9 protein, 150 pmol of each chemically modified synthetic 3’-sgRNA and 5’-sgRNA. *(7)* RNP-based CRISPR-Cas9 facilitated HDR with competing templates: 80 pmol purified Cas9 protein, 300 pmol chemically modified synthetic sgRNAs, 50 pmol ssODN HDR template carrying PD mutation and 50 pmol ssODN HDR template carrying a synonymous mutation. *(8)* RNA-based prime editing: 4 μg in vitro transcribed nCas9-RT mRNA, 100 pmol chemically modified synthetic pegRNA (IDT or Synthego) and 50 pmol chemically modified synthetic ngRNA (Synthego). Detailed protocols can be found on protocols.io (dx.doi.org/10.17504/protocols.io.e6nvwkkewvmk/v2).

#### Editing pipelines

We used two different editing pipelines termed *Pipeline A* and *Pipeline B* to generate the iSCORE-PD collection (Figure 3A,B). The editing pipeline used to create each cell line in the iSCORE-PD collection is included in Supplemental Table 3.

*Pipeline A* utilizes FACS-enrichment post-nucleofection to purify effectively transfected cells, followed by clonal expansion and genotyping to allow the isolation of clonal, correctly targeted lines (estimated time for the editing pipeline is 30–35 days). Following nucleofection, the hESCs are plated on MEFs in 10 μM ROCK inhibitor (Y27632, ToCris) containing hESC media (previously described in hPSC culture section) at high density (1 nucleofection/1 well 6 well plate). 48–72 h after nucleofection, Accutase-dissociated single cells are FACS-sorted for the expression of the respective fluorescent marker protein and either directly used for bulk NGS based validation of the desired genome modification or subsequently plated at clonal density (250 to 350 cells/cm2) on MEFs in hESC media supplemented with 10 μm ROCK inhibitor (Y27632, ToCris) for the first 24 hr. Individual colonies are picked and grown 7 to 14 days after electroporation. Correctly targeted clones were subsequently identified by Sanger or NGS sequencing. A detailed protocol can be found on protocols.io (dx.doi.org/10.17504/protocols.io.b4piqvke).

*Pipeline B* (high throughput hPSCs genome editing) involves low cell number nucleofection, limited dilution, and NGS-dependent genotyping to identify desirable edits in a 96-well plate system. This integrated workflow allows the efficient isolation of correctly edited clonal cell lines, even at low frequency, within a shorter time frame compared to previous approaches (21–35 days). As described previously^40^, the nucleofected cells are directly seeded onto MEFs in 96-well plates, at seeding densities of 1000 cells/plate in hPSCs media containing 10 μm ROCK inhibitor (Y27632, ToCris). After individual colonies appear around day 14, plates are duplicated for *(1)* maintenance and *(2)* DNA extraction for NGS-based identification of wells that contain cells with the desired genetic modification. To duplicate plates, cells are washed with PBS (Corning) and treated with 40μL 0.25% trypsin for 5 min at 37 C. 60μL hESC media containing 10 μM Rock inhibitor (Y27632, ToCris) is added to each well to inactivate trypsin. Cells are gently dissociated, and half (50 μL) of the cell suspension is reseeded to a new MEF containing 96-well plate pre-loaded with 100μL hPSC media containing 10 μM Rock inhibitor (Y27632, ToCris) and cultured for another 7 days with hPSC media.

#### NGS-based identification of validation of targeted clonal lines

50μL of cell suspension/well obtained during plate duplication is transferred to a 96-well PCR plate pre-loaded with 50μL 2X lysis buffer (100mM KCl, 4mM MgCl2, 0.9% NP-40, 0.9% Tween-20, 500μg/mL proteinase K, in 20mM Tris-HCl, pH 8) for DNA extraction (50 C overnight incubation followed by 95 C 10 min [proteinase K inactivation]). A ~300bp genomic region covering the designed mutation is amplified (Supplemental table 5) containing NGS barcode attachment sites (GCTCTTCCGATCT) from 2ul cell lysis from each well with Titan DNA polymerase. Amplicons were purified at the UC Berkeley DNA Sequencing Facility, then i5/i7 barcoded in indexing PCR, pooled and sequenced on 150PE iSeq in the NGS core facility at the Innovative Genomics Institute (IGI). CRISPResso2^139^ in prime editing mode was used to analyze the NGS data to identify wells containing the designed mutation, with the following criteria. Heterozygous candidates: number of reads aligned >100, 70% >mutant allele frequency >20%, indels frequency <5%; homozygous candidates: number of reads aligned >100, mutant allele frequency >70%, indels frequency <5%. Wells containing the desired editCells in those identified wells were single cell subcloned once and genotyped clonally to confirm cell line purity to ensure clonality. Detailed protocols for high throughput hPSCs genome editing (dx.doi.org/10.17504/protocols.io.b4mmqu46) and genotyping by next generation sequencing https://doi.org/10.17504/protocols.io.b4n3qvgn) can be found on protocols.io. For clarity, NGS results reported in any of the figures of this publication showcase only representative reads. Any NGS reads below 1% of the total result were removed. The full NGS report can be found with the rest of raw data files (10.5281/zenodo.10511722[1] /AMP-PD data repositories).

### Zygosity confirmation by SNP detection

The SNP closest to the editing site for each genetic edit was identified from the whole genome sequencing data of parental WIBR3 hESCs. A genome DNA region flanking the SNP and the editing site was amplified by PCR (Supplemental Table 5) and sequenced by Sanger sequencing or NGS. Clones showing LOH were removed from the final collection.

### Cortical spheroid differentiation

hESCs were differentiated into early cortical spheroids following an adaptation of a published protocol^140,141^. In brief, hESC colonies were dissociated and plated into pre-coated 6-well Aggrewell 800 plates at a concentration of 18M cells per well in hESC media with 10 μM Rock Inhibitor (ToCris). The next day (Day 1), the aggregates were removed, sedimented, and added to an ultralow adherence plate with hESC media supplemented with 5 μM Dorsomorphin (SelleckChem) and 10 μM SB431542 (SelleckChem) (media changed daily).. On Day 6, media was replaced with Neural Precursor Expansion Media (Neurobasal medium + B27 supplement without vitamin A (2% vol/vol) + Penicillin-Streptomycin (100U/ml) + GlutaMAX (1% vol/vol) + HEPES Buffer (1% vol/vol) + FGF2 (20 ng/ml) + EGF (20 ng/ml)) (media is changed every day until Day 16 and then every other day until Day 25). For specific details consult published materials on protocols.io: https://doi.org/10.17504/protocols.io.5jyl8po57g2w/v1.

### Cell irradiation

hESCs were transferred from MEFs to feeder-free matrigel substrate with conditioned media for 2 weeks previous to this experiment. hESCs at 50% confluence or cortical spheroids on day 25 of differentiation were irradiated at 0, 0.5, 2, 5, and 10 Gy using a discrete cesium source. 24 hours post irradiation, cells were collected and dissociated for MULTI-Seq barcoding and sequencing. For specific details consult published materials on protocols.io (https://doi.org/10.17504/protocols.io.bp2l6xwbzlqe/v1).

### MULTI-Seq Barcoding and Single-Cell Library Preparation of Irradiated Samples

#### hESCs:

Each irradiation condition was labeled with a lipid-modified barcoded MULTI-seq oligo following a previously described protocol^142^. In short, cells in PBS were incubated with a 1:1 molar ratio of lipid-modified Anchor Oligo:Barcode Oligo for 5 min on ice. Then an equimolar amount of lipid-modified co-anchor was added for an additional 5 min incubation on ice. Then cells were washed twice with ice cold PBS (Corning) + 1%BSA (Fisher) to sequester the anchor oligos, strained, counted, and pooled for single-cell sequencing. 10x single-cell RNA sequencing was performed according to manufacturer’s instructions using the Chromium Single Cell 3ʹ Reagent Kits v3 with Feature Barcoding Technology. For specific details consult published materials on protocols.io (https://doi.org/10.17504/protocols.io.kxygx3xzkg8j/v1.) Deconvolution of MULTI-Seq barcodes was performed as described previously^142^ using the MULTI-seq package at https://github.com/chris-mcginnis-ucsf/MULTI-seq/.

#### Cortical Spheroids:

single-cell suspensions were FACS-sorted to remove debris and aggregates, then 10x single-cell RNA sequencing was performed according to manufacturer’s instructions using the Chromium Single Cell 3ʹ Reagent Kits v3, targeting 2,000 cells per irradiation condition using one 10x lane per condition. Single-cell analysis of all irradiated samples was performed using Seurat v4^143^ according to default parameters for normalization and integration of data sets. Droplets with more than 15% mitochondrial reads detected were excluded as poor analysis candidates due to likelihood of cell death resulting in poor RNA representation. Plots were generated using ggplot2 in R.

### Dopaminergic neuron differentiation

Feeder-free adapted WIBR3 hESCs were differentiated into dopaminergic neurons as per previously reported protocols with slight modifications^54,55^. Briefly, hESC colonies were dissociated into single cells and seeded onto matrigel coated plates at a density of 400–600k cells per well of a 6 well plate in mTeSR (Stem Cell Technologies) containing 10 μM Rock inhibitor (Y27632, ToCris). Differentiation was induced sequentially with media A - 3 days (Neurobasal media (Gibco) + N2 supplement (Gibco; 1% vol/vol) + B27 supplement without vitamin A (Gibco; 2% vol/vol) + L-Glutamine (Gibco; 2 mM) + Penicillin-Streptomycin (Gibco; 100U/ml) + SHH C25II (R&D systems; 100–200 ng/ml) + CHIR99021 (ToCris; 0.7 μM) + LDN (Stemgent; 250 nM) + SB431542 (SelleckChem; 10 μM)), B −3 days (Neurobasal media (Gibco) + N2 supplement (Gibco; 1% vol/vol) + B27 supplement without vitamin A (Gibco; 2% vol/vol) + L-Glutamine (Gibco; 2 mM) + Penicillin-Streptomycin (Gibco; 100U/ml) + SHH C25II (R&D Systems; 100–200 ng/ml) + CHIR99021 (ToCris; 7.5 μM) + LDN (Stemgent; 250 nM) + SB431542 (SelleckChem; 10 μM)), C - 3 days (Neurobasal media (Gibco) + N2 supplement (Gibco; 1% vol/vol) + B27 supplement (Gibco; 2% vol/vol) + L-Glutamine (Gibco; 2 mM) + Penicillin-Streptomycin (Gibco; 100U/ml) + CHIR99021 (SelleckChem; 7.5 μM)) and D −1 day (Neurobasal media (Gibco) + B27 supplement (Gibco; 2% vol/vol) + L-Glutamine (GIbco; 2 mM) + Penicillin-Streptomycin (Gibco; 100U/ml) + BDNF (PeProtech; 20 ng/ml) + GDNF (PeProtech; 20 ng/ml) + Ascorbic acid (Sigma; 200 μM) + Dibutyryl-cAMP (SelleckChem; 0.5 mM) + TGFβ3 (R&D Systems; 1 ng/ml) + CHIR99021 (SelleckChem; 3 μM)) over an 10 day period. On day 11, cells were dissociated and plated (1:2 ratio) at high density and maintained in maturation media (Neurobasal media (Gibco) + B27 supplement (Gibco; 2% vol/vol) + L-Glutamine (GIbco; 2 mM) + Penicillin-Streptomycin (Gibco; 100U/ml) + BDNF (PeProtech; 20 ng/ml) + GDNF (PeProtech; 20 ng/ml) + Ascorbic acid (Sigma; 200 μM) + Dibutyryl-cAMP (SelleckChem; 0.5 mM) + TGFβ3 (R&D Systems; 1 ng/ml) + DAPT (ToCris; 10 μM)) until day 16, when they were replated at the similar high density in 12 well plate and left to mature until day 24. On day 25, cells were dissociated for the final time with accutase and replated at no less than 1–2 × 10^6^ cells per well of 12 well plate and left to mature until post-differentiation experiments were carried out. For specific details consult published materials on protocols.io: https://doi.org/10.17504/protocols.io.3byl4q8yovo5/v1.

### scRNA-Seq of dopaminergic neurons - 10x Genomics library preparation

Dopaminergic neurons were harvested with Accutase on day 35, 36 or 37 of culture and subsequently labeled with 10x Genomics CellPlex reagents, following the manufacturer recommendation (10x Genomics CG000391 Rev B). After labeling with Cell Multiplexing Oligos (CMOs), samples were pooled and taken for 10x Genomics library preparation, following manufacturer recommendations with target capture of 30,000 cells per 10x lane (Chromium Single Cell 3’ Reagent Kits v3.1, User Guide CG000388 Rev C).

### scRNASeq of dopaminergic neurons – Data analysis

After next-generation sequencing of 10x Genomics libraries (NovaSeq 6000), FASTQ files were processed with 10x Genomics CellRanger pipeline (v7.0.1) to demultiplex and generate count matrices for each sample. Data for each sample were first filtered to remove low-quality cells (cells with fewer than 1,500 genes detected, greater than 30,000 RNA counts, and greater than 10% mitochondrial reads were removed). Filtered datasets were each processed individually with Seurat v4^143^, using the SCTransform function for normalization and variance stabilization. Integration of the SCTransformed data was performed to generate a combined dataset of 10,097 cells.

### Microglia differentiation

To generate in vitro differentiated microglia cells (iMGs), we adapted a previously published protocol^60^. Undifferentiated feeder free hESC colonies maintained in mTeSR (Stem Cell Technology) were seeded at low density into cell culture flasks coated with reduced growth factor matrigel (30 colonies/T75 flask (Fisher)) using manual passaging. In vitro differentiation was achieved by sequential culture of the cells in the following media: Step 1 (mTeSR (Stem Cell Tech) + 80 ng/ml BMP4 (PeProtech) - 4 days), Step 2 (StemPro-34 SFM (Gibco) + 2 mM GlutaMAX (Gibco) + 80 ng/ml VEGF (PeProtech), 25 ng/ml FGF (PeProtech) + 100 ng/ml SCF (PeProtech) - 3 days), Step 3 (StemPro-34 SFM (Gibco) + 2 mM GlutaMAX + 50 ng/ml SCF (PeProtech) + 50 ng/ml IL-3 (PeProtech) + 5 ng/ml TPO (PeProtech) + 50 ng/ul M-CSF (PeProtech) + 50 ng/ul Flt3 (PeProtech) - 9 days) and Step 4 (StemPro-34 SFM (Gibco) + 2 mM GlutaMAX + 50 ng/ml M-CSF (PeProtech) + 50 ng/ml Ftl3 (PeProtech) + 25 ng/ml GM-CSF (PeProtech) - 14 days). After ~28 days, microglia progenitors are ready to be isolated and plated on Primaria plates (Corning) for maturation (at least 2 weeks) in microglia maturation media (Neurobasal media (Gibco) + N2 Neuroplex (Gemini; 1x final concentration) + GEM21 Neuroplex (Gemini; 1x final concentration) + 20% AlbuMAX I (Gibco; 0.2% final concentration) + NaCl (Fisher; 5M) (50mM final concentration) + sodium pyruvate 100x (Gibco’ 1x final concentration) + glutaMAX 100x (Gibco; 1x final concentration) + Penicillin-Streptomycin (Gibco; 100U/ml) + 50 ng/ml TGF-β1 (PeProtech) + 100 ng/ml IL-34 (PeProtech) + 12.5 ng/ml M-CSF (PeProtech)). Detailed protocols for microglial differentiation can be found on protocols.io (https://doi.org/10.17504/protocols.io.4r3l22zbjl1y/v1). Microglia cells were evaluated by immunocytochemistry (https://doi.org/10.17504/protocols.io.yxmvm3146l3p/v1) and FACS-based analysis (https://doi.org/10.17504/protocols.io.81wgbxokqlpk/v1) in order to confirm expression of precursor and mature microglia markers (CD16, CD45, CX3CR1, P2RY12, CD11b, CD14, IBA1, PU.1). FACS data was analyzed using FlowJo software (version 10.8.0).

### Immunocytochemistry

Immunocytochemistry was used to assess biomarker expression to characterize each of the cell types shown in this publication. Briefly, samples were fixed in PFA and permeabilized (0.03% triton when necessary) and blocked (BSA or serum) as required depending on the biomarker being analyzed on hESCs (OCT4 (DSHB), SSEA4 (DSHB)) or our differentiated cell cultures: dopaminergic neurons (TH (Pelfreeze), FOXA2 (R&D Systems)) or microglia (IBA1 (Abcam), P2RY12 (Sigma), CX3XR1 (Biolegend), PU.1 (Cell Signaling Tech)). Fluorochrome conjugated secondary antibodies were used to image our samples in an epifluorescence or confocal microscope. Alkaline phosphatase activity was measured using Vector^®^ Black Substrate Kit, Alkaline Phosphatase (Vector Laboratories). Specific details on the protocol used can be found in protocols.io (https://doi.org/10.17504/protocols.io.yxmvm3146l3p/v1). For specific details on our staining of pluripotency markers in our hESCs consult: https://doi.org/10.17504/protocols.io.b4yyqxxw. OCT4, SSEA4 and AP Images from our hESC cultures were captured using a 10X objective on a fluorescence microscope (Zeiss ZEN 3.8).. Magnification may differ depending on which microscope-camera set was used to capture the images. This was a result of which team within the collaboration was in charge of generating a specific cell line and its analysis through the QC steps.

### RNA Isolation and qRT-PCR

Total RNA was isolated from cell pellets with the RNeasy kit (Qiagen). 1–2μg of RNA was used for cDNA synthesis using High-capacity reverse transcriptase kit (ThermoFisher Scientific). Real-time qRT-PCR was performed on the QuantStudio 6 Flex thermocycler using PowerUp SYBR green master mix (ThermoFisher Scientific). All reactions were prepared according to the manufacturer instructions. Results were normalized to GAPDH and compared against human fibroblast samples (MRC-9, BJ1-hTERT and GM01660). All primer sequences used for qRT-PCR were listed in Supplemental Table 5. For a detailed protocol consult: https://doi.org/10.17504/protocols.io.4r3l22r9pl1y/v1. Plots were generated using GraphPad Prism (version 10.1.2 [324]).

### Southern blot

Southern blotting was performed following standard protocols (https://cshprotocols.cshlp.org/content/2021/7/pdb.top100396#cited-by) to validate the structural integrity and exclude the loss of heterozygosity (LOH) at a genomic locus of interest (PRKN, DJ1, FBXO7 and SYNJ1) resulting from CRISPR/Cas9 or prime editing-based genome editing experiments in hESCs. Southern blot probes were generated by PCR amplification (AccuPrime^™^ Taq DNA Polymerase, high fidelity (ThermoFisher)) of a 150bp to 600bp large genomic region 3’- and 5’ to the targeted genomic region. Southern blot probes were radiolabeled using the Prime-it Random Primer Labeling Kit (Agilent) according to the manufacturer’s instructions. Restriction digested genomic DNA isolated from clonally expanded genome edited hESC lines was separated on a 0.8% agarose (Sigma) gel, transferred to a nylon membrane (Amersham), and hybridized with 32P random primers labeled southern blot probes. Oligonucleotide sequences and restriction enzyme information can be found in Supplemental Table 5. Detailed protocols for southern blot analysis can be found on protocols.io (https://doi.org/10.17504/protocols.io.bp2l6xe6dlqe/v1).

## Supplementary Material

Supplement 1 **Supplemental Table 1**. List of structural variants in parental WIBR3 hESC line.

Supplement 2 **Supplemental Table 2.** List of coding variants in parental WIBR3 hESC line.

Supplement 3 **Supplemental Table 3.** Full description of all cell lines included in iSCORE-PD cell collection (control wild type, edited wild-types, SNCA A53T and A30P, PRKN X3DEL, PINK1 Q129X, DJ1/PARK7 X1-5DEL, LRRK2 G2019S, ATP13A2 FS, FBXO7 R498X/FS, DNAJC6 c.801–2 A>G/FS, SYNJ1 R258Q/FS, VPS13c A444P/FS and W395C/FS and GBA1 IVS2+1 G>A/FS). Details include: Full cell line name, gene targeted, editing strategy, locus genetic description, rs-number, chromosome:basepair (hg38), allelic dosage, DNA annotation, predicted protein, Cellosaurus ID, pipeline (A/B).

Supplement 4 **Supplemental Table 4.** Quality control overview for iSCORE-PD cell line generation.

Supplement 5 **Supplemental Table 5.** Oligonucleotide sequence collection used for genome engineering and QC steps. Primer sequences for Sanger and NGS genotyping, HDR oligonucleotide sequences, pegRNA sequences, sgRNA sequences, TALEN arm sequences, southern blot probe sequences and primers for SNP detection (zygosity evaluation).

1 Supplemental Figure 1. Characterization of WIBR3 in feeder free conditions.
(A) Immunocytochemistry for pluripotency markers OCT4 (green) and SSEA4 (red) on hESC colonies in feeder free cultures from parental WIBR3 hESC and the clonal lines WIBR3-S1, WIBR3-S2 and WIBR3-S3. Scale bar 100 μm.
(B) The percentage of genetic variant types present in WIBR3 grouped by their predicted consequences on coding sequences.
Supplemental Figure 2. Dopaminergic neuron immunostaining and gene expression analysis.
(A) Immunofluorescence of TH and FOXA2 in dopaminergic neurons (at day 35) derived from three subclones (S1, S2, S3) of WIBR3, scale bar 100 μm.
(B) qRT-PCR quantification of midbrain floor plate progenitor and dopaminergic neuron markers at day 11 and day 25 of differentiation of WIBR3 hESC (dark grey) and KOLF2.1 ihPSCs (light grey). Relative gene expression is calculated relative to the expression of GAPDH. (N = 3; MEAN ± SD).
Supplemental Figure 3. Quality control metrics of single cell data.
(A) UMAPs of WIBR3 dopamine neurons of three subclones, to visualize distribution of cells in each cluster from an integrated seurat single cells dataset.
(B) Heatmap showing top 10 genes differentially expressed in each cluster.
(C) Quality control plots of nCount RNA reads (UMIs) and feature RNA reads (genes) for each subclone.
(D) UMAP feature plots showing expression patterns across cell clusters of floor plate progenitor and dopamine neuron specific marker genes.
Supplemental Figure 4. scRNASeq comparison of WIBR3 cells to the FOUNDIN-PD reference.
(A) *(i)* UMAP plot of all 10,097 WIBR3 cells profiled, with labels representing a cell’s assignment to a corresponding cell type as defined in the FOUNDIN-PD reference dataset. Labels for each cell are identified using the SingleR classifier in R (Wilcoxon rank sum test). *(ii)* Stacked barplot depicting the proportion of cell type identities across the entire 10,097 cell WIBR3 dataset or the 416,216 cells in the FOUNDIN-PD reference dataset. *(iii)* UMAP of the 416,216 cells in the FOUNDIN-PD reference dataset, with the author’s default cell type labels applied.
(B) Heatmap depicting the cell type classification of 10,097 WIBR3 cells (rows) into a list of cognate cell type labels as defined in the FOUNDIN-PD reference dataset. Plotted values represent normalized cell type assignment scores as calculated by the SingleR package in R (Wilcoxon rank-sum test). Any cells retaining undefined identities after SingleR classification (“NA”) are omitted from downstream analyses. All 10,097 WIBR3 cells depicted in the UMAP space of the FOUNDIN-PD dataset shown in panel *A iii*, with each cell depicted with a FOUNDIN-PD cell type label applied by SingleR.
(D) Heatmap of WIBR3 cells (columns) grouped by FOUNDIN-PD labels as applied by SingleR. Rows depict the top 10 marker genes that define the WIBR3 cells assigned to each FOUNDIN-PD cell type identity. Markers lists are identified with the “FindAllMarkers” function in Seurat, using the “MAST” test.
Supplemental Figure 5. In vitro differentiation of microglia from WIBR3-S2 and WIBR3-S3
(A–D) Representative phase contrast (A) and immunostaining (B–C) images of in vitro differentiated microglia derived from subclone WIBR-S2 for microglia-specific markers IBA1, P2RY12, and CX3CR1 (terminal diff day 14).
(E–H) Representative phase contrast (E) and immunostaining (F–H) images of in vitro differentiated microglia derived from subclone WIBR-S3 for microglia-specific markers IBA1, P2RY12, and CX3CR1 (terminal diff day 14). Scale bar (phase contrast): 50 μm; Scale bar (ICC): 10 μm.
Supplemental Figure 6. Characterization of edited wild type cell lines.
Immunocytochemistry of edited wild-type (EWT) cells lines for pluripotency markers OCT4 (green), SSEA4 (red) and alkaline phosphatase (black). EWT refers to cell lines that have undergone the editing pipelines, were not genetically modified and remain genotypically wild type. WIBR3_EWT_S1–3 were isolated from a prime editing experiment using Pipeline B, WIBR3_EWT_S4–5 were isolated from CRISPR/Cas9-facilitated HDR experiments using Pipeline B and WIBR3_EWT_S6–8 were isolated from a prime editing experiment using Pipeline A. Scale bar 100 μm.
Supplemental Figure 7. Genome editing and quality control of WIBR3 hESCs carrying PD-associated mutations in SNCA.
(A) Targeting strategy to generate SNCA A53T mutation using prime editing.
(B) NGS-based genotyping to confirm correct editing for SNCA A53T in clones WIBR3_SNCA_A53T_1, WIBR3_SNCA_A53T_2 and WIBR3_SNCA_A53T_4. Bold bases indicate base substitute.
(C) Immunocytochemistry of hESC cultures for pluripotency markers OCT4 (green), SSEA4 (red), and alkaline phosphatase (black). Scale bar 100 μm.
(D) Targeting strategy to generate SNCA A30P mutation by prime editing.
(E) NGS-based genotyping to confirm correct editing for SNCA A30P in clones WIBR3_SNCA_A30P_A2–3, WIBR3_SNCA_A30P_Homo_C8–2, WIBR3_SNCA_A30P_E1–3 and WIBR3_SNCA_A30P_F12–1. Bold bases indicate base substitution. The generation of the WIBR3_SNCA_A30P cell lines was already reported^61^.
(F) Immunocytochemistry of hESC cultures for pluripotency markers OCT4 (green), SSEA4 (red) and alkaline phosphatase (black). Scale bar 100 μm.
(G) Zygosity analysis using Sanger sequencing to detect heterozygous SNP to exclude LOH in clone WIBR3_SNCA_A30P_Homo_C8–2.
Supplemental Figure 8. Genome editing and quality control of WIBR3 hESCs carrying PD-associated deletions in PRKN.
(A) Schematic illustrating targeting strategy to generate PRKN Ex3del mutation by CRISPR/Cas9 dual guide strategy. Included are genomic location of sgRNAs, Southern blot (SB) probes and restriction enzymes used for southern blot.
(B) NGS-based genotyping to confirm correct editing for PRKN Ex3del in clones WIBR3_PRKN_X3DEL_B1–3, WIBR3_PRKN_X3DEL_F2–5 and WIBR3_PRKN_X3DEL_H2–2 (deletion junction). Red box indicates single base insertion.
(C) Immunocytochemistry of hESC cultures for pluripotency markers OCT4 (green), SSEA4 (red) and alkaline phosphatase (black). Scale bar 100 μm.
(D, E) Southern blot analysis of WIBR3_PRKN_X3DEL cell lines to excluded LOH. Genomic DNA was digested with indicated enzymes and hybridized with 3’ and 5’ probe indicated in (A). Expected fragment size for wild type and PRKN_X3DEL allele are indicated for each digest. This analysis indicates that the clone WIBR3_PRKN_X3DEL_B1–3 carries a larger deletion on one allele.
(F) NGS analysis of the new deletion junction in the clone WIBR3_PRKN_X3DEL_B1–3.
Supplemental Figure 9. Genome editing and quality control of WIBR3 hESCs carrying PD-associated mutations in PINK1.
(A) Schematic illustrating targeting strategy to generate PINK1 Q129X mutation by CRISPR/Cas9 facilitated HDR.
(B) NGS-based genotyping to confirm correct editing for PINK1 Q129X in clones WIBR3_PINK1_Q129X_C4–1, WIBR3_PINK1_Q129X_E2–2 and WIBR3_PINK1_Q129X_E7–1. Bold bases indicate base substitution.
(C) Immunocytochemistry of hESC cultures for pluripotency markers OCT4 (green), SSEA4 (red) and alkaline phosphatase (black). Scale bar 100 μm.
(D) Zygosity analysis using Sanger sequencing to detect heterozygous SNP to exclude LOH in any of the PINK1 Q129X clones.
Supplemental Figure 10. Genome editing and quality control of WIBR3 hESCs carrying PD-associated deletions in PARK7/DJ1.
(A) Schematic illustrating targeting strategy to generate PARK7/DJ1 Ex1–5del mutation by CRISPR/Cas9 dual guide strategy. Included are genomic location of sgRNAs, Southern blot (SB) probes and restriction enzymes used for southern blot.
(B) NGS-based genotyping to confirm correct editing for PARK7/DJ1 Ex1–5del in clones WIBR3_DJ1_X1–5DEL_2860, WIBR3_DJ1_X1–5DEL_2872 and WIBR3_DJ1_X1–5DEL_2876 (deletion junction). “-“ indicates base deletion.
(C) Immunocytochemistry of hESC cultures for pluripotency markers OCT4 (green), SSEA4 (red), and alkaline phosphatase (black). Scale bar 100 μm.
(D, E) Southern blot analysis of homozygous and heterozygous WIBR3_DJ1_X1–5DEL cell lines to excluded LOH. Only homozygous cell lines WIBR3_DJ1_X1–5DEL_2860, WIBR3_DJ1_X1–5DEL_2872 and WIBR3_DJ1_X1–5DEL_2876 are included in iSCORE-PD collection. Genomic DNA was digested with indicated enzymes and hybridized with 5’ probes A and B indicated in (A). Expected fragment size for wild type and DJ1_X1–5DEL alleles are indicated for each digest.
Supplemental Figure 11. Genome editing and quality control of WIBR3 hESCs carrying PD-associated mutations in LRRK2.
(A) Schematic illustrating targeting strategy to generate LRRK2 G2019S mutation by prime editing and TALEN or CRISPR/Cas9 facilitated HDR.
(B) NGS-based genotyping to confirm correct editing for LRRK2 G2019S in clones WIBR3_LRRK2_G2019S_5_Het, WIBR3_LRRK2_G2019S_6_Het, WIBR3_LRRK2_G2019S_65_Homo, WIBR3_LRRK2_G2019S_216_Het and WIBR3_LRRK2_G2019S_2093_Het. Bold bases indicate base substitution. The generation of the WIBR3_LRRK2_G2019S_5_Het and WIBR3_LRRK2_G2019S_6_Het cell lines was already reported^61^.
(C) Immunocytochemistry of hESC cultures for pluripotency markers OCT4 (green), SSEA4 (red) and alkaline phosphatase (black). Scale bar 100 μm.
(D) Zygosity analysis using Sanger sequencing to detect heterozygous SNP to exclude LOH in cell line WIBR3_LRRK2_G2019S_65_Homo.
Supplemental Figure 12. Genome editing and quality control of WIBR3 hESCs carrying PD-associated alterations in ATP13A2.
(A) Schematic illustrating targeting strategy to generate CRISPR/Cas9 mediated frameshift mutation in ATP13A2.
(B) NGS-based genotyping to confirm a frameshift mutation in ATP13A2 in clones WIBR3_ATP13A2_FS_Homo_2_5, WIBR3_ATP13A2_FS_5_6, WIBR3_ATP13A2_FS_Homo_6_1, WIBR3_ATP13A2_FS_Homo_12_2 and WIBR3_ATP13A2_FS_Homo_12_6. Red box indicates base insertion. “-“ indicates base deletion.
(C) Immunocytochemistry of hESC cultures for pluripotency markers OCT4 (green), SSEA4 (red) and alkaline phosphatase (black). Scale bar 100 μm.
(D) Zygosity analysis using NGS sequencing to detect heterozygous SNP to exclude LOH in all ATP13A2 clones.
Supplemental Figure 13. Genome editing and quality control of WIBR3 hESCs carrying PD-associated alterations in FBXO7.
(A) Schematic illustrating targeting strategy to generate CRISPR/Cas9-mediated frameshift mutation in FBXO7. Included are genomic location of sgRNA, Southern blot (SB) probe and restriction enzymes used for southern blot.
(B) NGS-based genotyping to confirm a frameshift in mutation in FBXO7 in clone WIBR3_FBXO7_FS_A3–1. “-“ indicates base deletion.
(C) Immunocytochemistry of hESC cultures for pluripotency markers OCT4 (green), SSEA4 (red) and alkaline phosphatase (black). Scale bar 100 μm.
(D, E) Southern blot analysis of homozygous WIBR3_FBXO7_FS_A3_1 cell line to excluded LOH. Genomic DNA was digested with indicated enzymes and hybridized with 5’ probe indicated in (A). Expected fragment size for wild type and FBXO7 frameshift alleles are indicated for each digest.
(F) Zygosity analysis using NGS sequencing to detect heterozygous SNP to exclude LOH in the WIBR3_FBXO7_FS_A3_1 cell line.
Supplemental Figure 14. Genome editing and quality control of WIBR3 hESCs carrying PD-associated alterations DNAJC6.
A) Schematic illustrating targeting strategy to generate DNAJC6 c.801–2 A>G/FS mutation by CRISPR/Cas9 facilitated HDR.
(B) NGS-based genotyping to confirm correct editing for DNAJC6 c.801–2 A>G and frameshift mutation in clones WIBR3_DNAJC6_c.801–2 A>G+FS/FS_G12–2 and WIBR3_DNAJC6_FS/FS_H10–1. “-“ indicates base deletion.
(C) Immunocytochemistry of hESC cultures for pluripotency markers OCT4 (green), SSEA4 (red) and alkaline phosphatase (black). Scale bar 100 μm.
Supplemental Figure 15. Genome editing and quality control of WIBR3 hESCs carrying PD-associated alterations SYNJ1.
(A) Schematic illustrating targeting strategy to generate SYNJ1 R258Q/FS mutation by CRISPR/Cas9 facilitated HDR.
(B) NGS-based genotyping to confirm correct editing for SYNJ1 R258Q and frameshift mutation in clones WIBR3_SYNJ1_R258Q_Homo_A5–1 and WIBR3_SYNJ1_R258Q/FS_E4–1. Bold bases indicate base substitution. Red box indicates base insertion.
(C) Immunocytochemistry of hESC cultures for pluripotency markers OCT4 (green), SSEA4 (red) and alkaline phosphatase (black). Scale bar 100 μm.
(D-F) Southern blot analysis of WIBR3_SYNJ1 cell lines to excluded LOH. Genomic DNA was digested with indicated enzymes and hybridized with probes indicated in (A). Expected fragment size for wild-type and SYNJ1 alleles are indicated for each digest.
Supplemental Figure 16. Genome editing and quality control of WIBR3 hESCs carrying PD-associated alterations VPS13C.
(A) Schematic illustrating targeting strategy to generate VPS13C A444P mutation by CRISPR/Cas9 facilitated HDR. Included are genomic location of the A444P mutation, sgRNA, ssODN template including synonymous ssODN mutations (SYN) in sgRNA-target site to prevent re-cutting of edited alleles.
(B) NGS-based genotyping to confirm correct editing for A444P and frameshift mutation in VPS13C in the clones WIBR3_VPS13C_A444P_Homo_C8–2, WIBR3_VPS13C_A444P_Het_E12–1 and WIBR3_VPS13c_FS_Homo_H3–1. Bold bases indicate base substitution. “-“ indicates base deletion.
(C) Immunocytochemistry of hESC cultures for pluripotency markers OCT4 (green), SSEA4 (red) and alkaline phosphatase (black). Scale bar 100 μm.
(D) Zygosity analysis using Sanger sequencing to detect heterozygous SNP to exclude LOH in clones WIBR3_VPS13C_A444P_Homo_C8–2 and WIBR3_VPS13C_A444P_Homo_H3–1.
(E) Schematic illustrating targeting strategy to generate VPS13C W395C and frameshift mutation by CRISPR/Cas9 facilitated HDR.
(F) NGS-based genotyping to confirm correct editing for VPS13C W395C and frameshift mutation in the clones WIBR3_VPS13C_W395C_Homo_C3–1, WIBR3_VPS13C_W395C_Homo_C6–2, WIBR3_VPS13C_W395C_Homo_C11–3 and WIBR3_VPS13C_FS_Homo_E10–2. Bold bases indicates base substitution. “-“ indicates base deletion.
(G) Immunocytochemistry of hESC cultures for pluripotency markers OCT4 (green), SSEA4 (red) and alkaline phosphatase (black). Scale bar 100 μm.
Supplemental Figure 17. Genome editing and quality control of WIBR3 hESCs carrying PD-associated alterations GBA1.
(A) Schematic illustrating targeting strategy to generate GBA1 IVS2+1 mutation by CRISPR/Cas9 facilitated HDR. To generate the heterozygous mutations a competing HDR template (ssODNs) containing synonymous mutations (SYN) in the sgRNA-target site was used.
(B) Sequences alignment of the wild type GBA1 gene (GBA1, WT), its pseudogene (GBAP1), the engineered GBA1 IVS2+1 mutation (GBA1, IVS2+1 G>A) and synonymous mutation (GBA1, A37=) in the targeted region. The mutated nucleotide and SNPs used to calculate the allelic balance between GBA1 and GBAP1 are highlighted in red boxes.
(C) NGS-based genotyping to confirm correct editing of the GBA IVS2+1 mutation and evaluate proper zygosity balance with GBAP1 for Clones WIBR3_GBA_IVS2_Het_3C3I, WIBR3_GBA_IVS2_Het_3C4B, WIBR3_GBA_FS_Het_10C2F, WIBR3_GBA_IVS2_E10B and WIBR3_GBA_IVS2_G2E. Bold bases indicate base substitution. Red box indicates base insertion.
(D) Immunocytochemistry of hESC cultures for pluripotency markers OCT4 (green), SSEA4 (red) and alkaline phosphatase (black). Scale bar 100 μm.

## Figures and Tables

**Table 1.** iSCORE-PD cell line collection summary (targeted genes, mutations generated, genetic inheritance, editing approach, number of cell lines and their genotype).

				CELLS IN COLLECTION	
╲	MUTATION	INHERITANCE	EDITING	MONOALLELIC - HETEROZYGOUS	BIALLELIC - HOMOZYGOUS
***SNCA (PARK1)***	A53T	Autosomal dominant	Prime editing	3	-
	A30P	Autosomal dominant	Prime editing	3	1
***PRKN (PARK2)***	EX3DEL	Autosomal recessive	CRISPR/Cas9 (dual guide)	-	3
***PINK1 (PARK6)***	Q129X	Autosomal recessive	CRISPR/Cas9 (HDR)	-	3
***DJ1 (PARK7)***	EX1–5DEL	Autosomal recessive	CRISPR/Cas9 (dual guide)	-	3
***LRRK2 (PARK8)***	G2019S	Autosomal dominant	Prime editing, TALEN and CRISPR/Cas9 (HDR)	4	1
***ATP13A2 (PARK9)***	FS	Autosomal recessive	CRISPR/Cas9 (HDR)	-	5
***FBXO7 (PARK15)***	R498X/FS	Autosomal recessive	CRISPR/Cas9 (HDR)	-	1
***DNAJC6 (PARK19)***	c.801–2 A>G/FS	Autosomal recessive	CRISPR/Cas9 (HDR)	-	2
***SYNJ1 (PARK20)***	R258Q/FS	Autosomal recessive	CRISPR/Cas9 (HDR)	-	2
***VPS13c (PARK23)***	W395C/FS	Autosomal recessive	CRISPR/Cas9 (HDR)	-	4
	A444P/FS	Autosomal recessive	CRISPR/Cas9 (HDR)	1	2
***GBA1***	IVS2+1/FS	Autosomal dominant^1^	CRISPR/Cas9 (HDR)	4	2
***Wild type***	-	-		Parental + 3 independent clones	
***Edited wild type (EWT)***	-	-	Prime editing	Prime editing (6) + CRISPR/Cas9 (2)	

1 mixed inheritance.

## Data Availability

All Genotyping and sequencing data is available at https://www.amp-pd.org/ via GP2 data sharing agreements This includes *(i)* Array genotyping data, *(ii)* Oxford Nanopore Technologies long-read sequencing *(iii)* Pacific Biosciences long-read sequencing, *(iv)* NGS and Sanger sequencing data for targeted genotyping and zygosity analysis, and *(v)* single cell RNA-seq data from dopaminergic neuron differentiation and p53 pathway analysis. All other materials can be found in the Zenodo repository: 10.5281/zenodo.10511722. This includes *(i)* aCGH reports, *(ii)* ICC images (microglia, DA neurons, hESC), *(iii)* southern blot raw film and gel images, *(iv)* qRT-PCR result files/analysis for dopaminergic neuron differentiation, and *(v)* FACS-analysis results for microglia.
