# Supplementary material for: iSCORE-PD: an isogenic stem cell collection to research Parkinson’s Disease
Source: bioRxiv. 2024 Feb 13:2024.02.12.579917. Preprint. [Version 1] doi: 10.1101/2024.02.12.579917 (PMC10888955; doi:10.1101/2024.02.12.579917)

Supplemental Figure 1, Busquets et al.

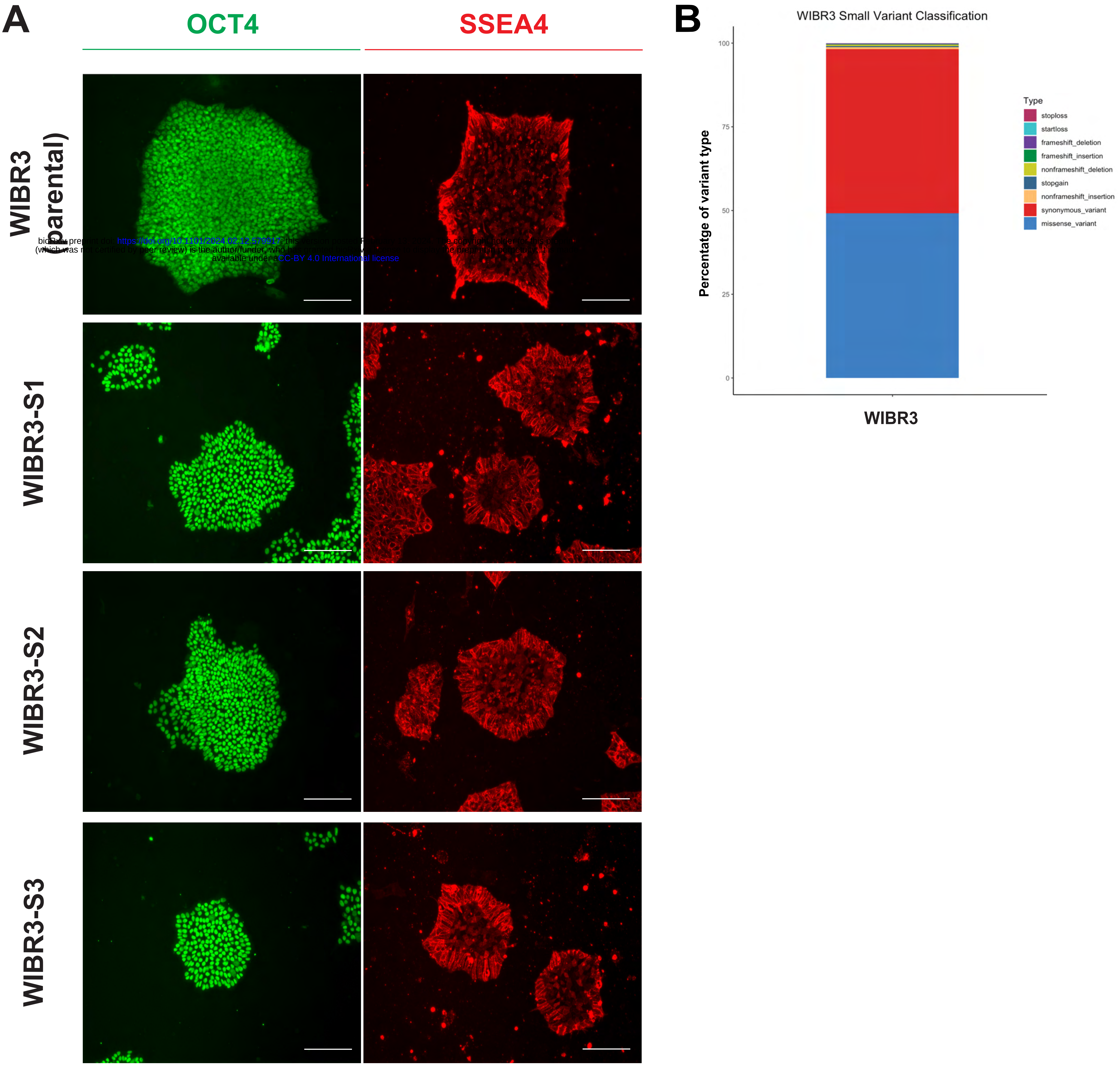

bioRxiv preprint doi: https://doi.org/10.1101/2024.02.12.579917; this version posted February 13, 2024. The copyright holder for this preprint (which was not certified by peer review) is the author/funder, who has granted bioRxiv a license to display the preprint in perpetuity. It is made available under aCC-BY 4.0 International license.

Supplemental Figure 2, Busquets et al.

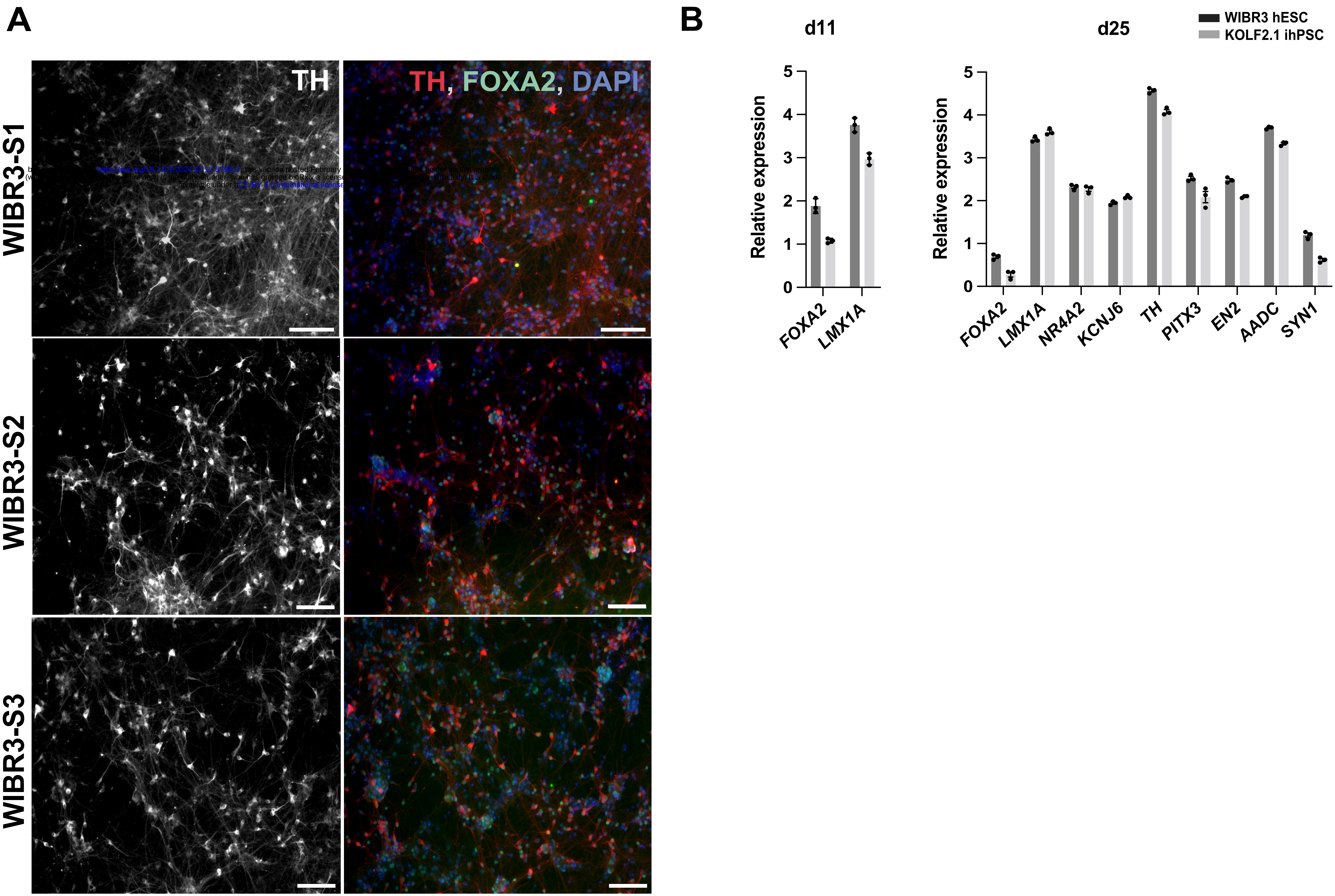

https://doi.org/10.1101/2024.02.12.579917; this version posted February [illegible] (which was not certified by peer review) is the author/funder, who has granted bioRxiv a license [illegible] available under aCC-BY 4.0 International license.

Supplemental Figure 3, Busquets et al.

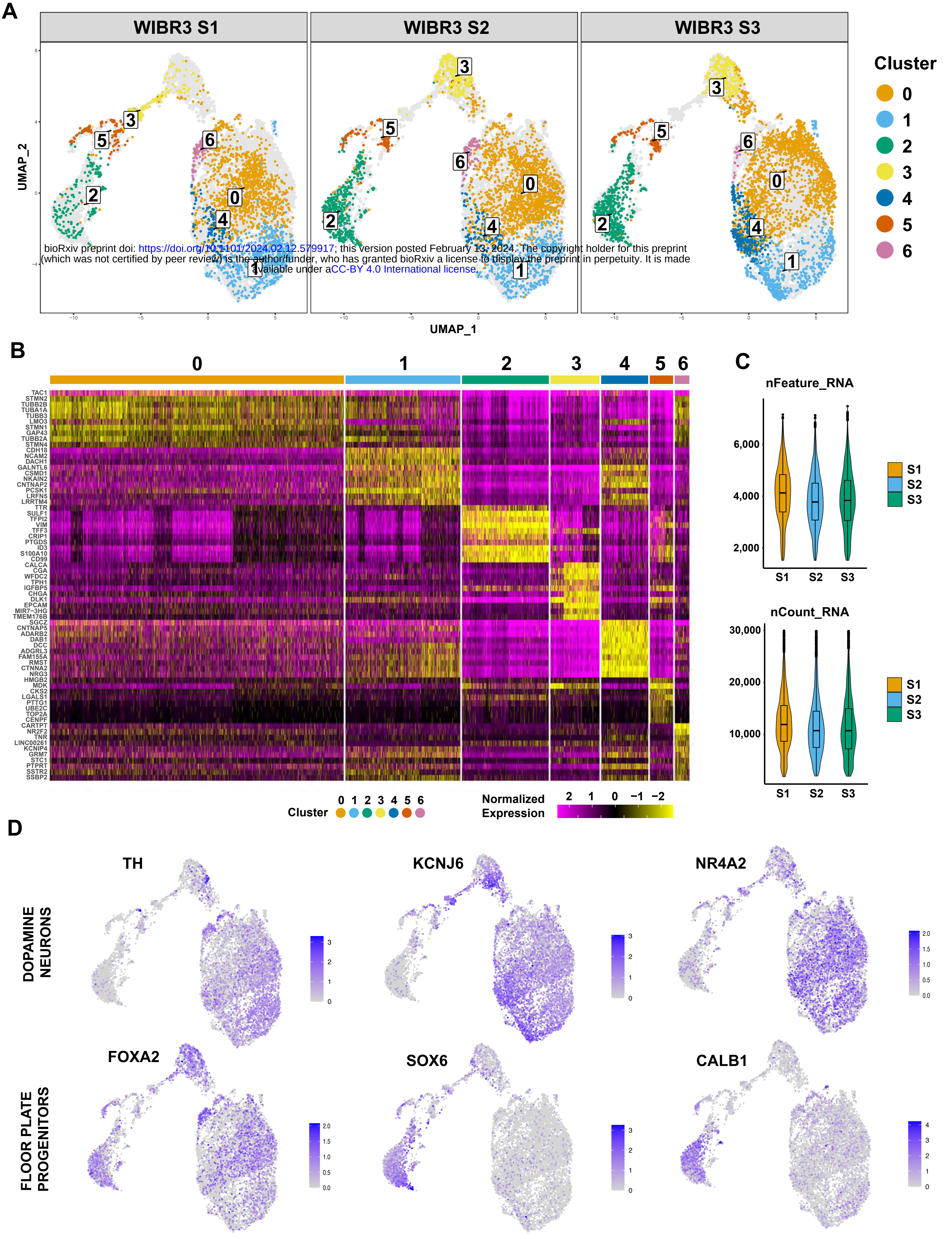

bioRxiv preprint doi: https://doi.org/10.1101/2024.02.12.579917; this version posted February 13, 2024. The copyright holder for this preprint (which was not certified by peer review) is the author/funder, who has granted bioRxiv a license to display the preprint in perpetuity. It is made available under aCC-BY 4.0 International license.

Supplemental Figure 4, Busquets et al.,

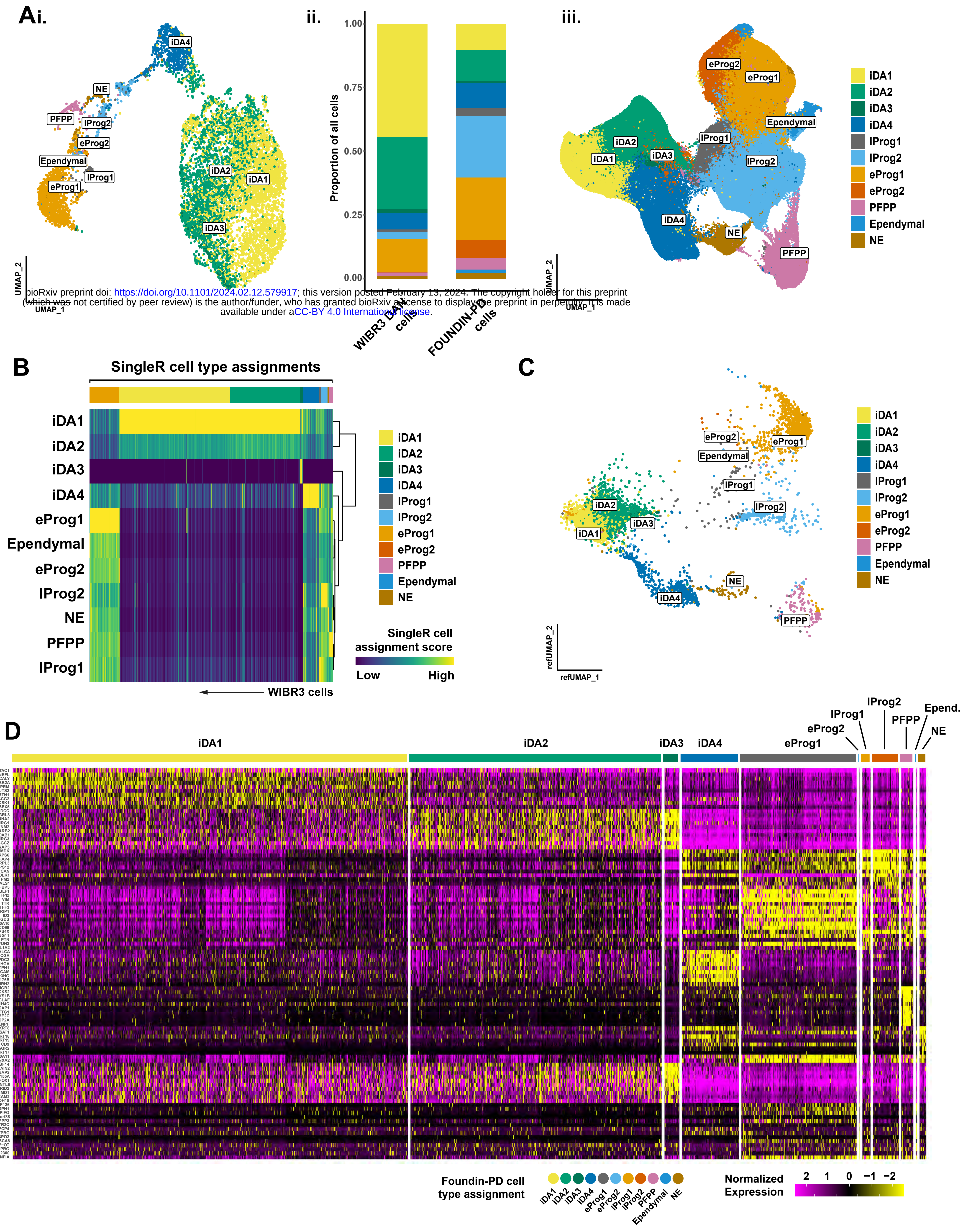

bioRxiv preprint doi: https://doi.org/10.1101/2024.02.12.579917; this version posted February 13, 2024. The copyright holder for this preprint (which was not certified by peer review) is the author/funder, who has granted bioRxiv a license to display the preprint in perpetuity. It is made available under aCC-BY 4.0 International license.

Supplemental Figure 5. Microglia ICC, Busquets et al.

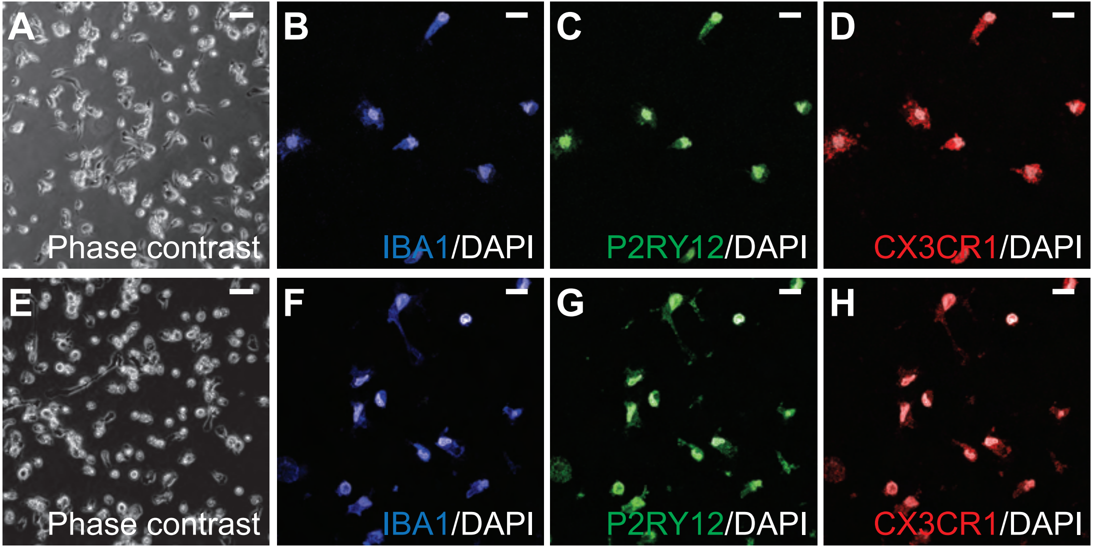

Supplemental Figure 6, Busquets et al.

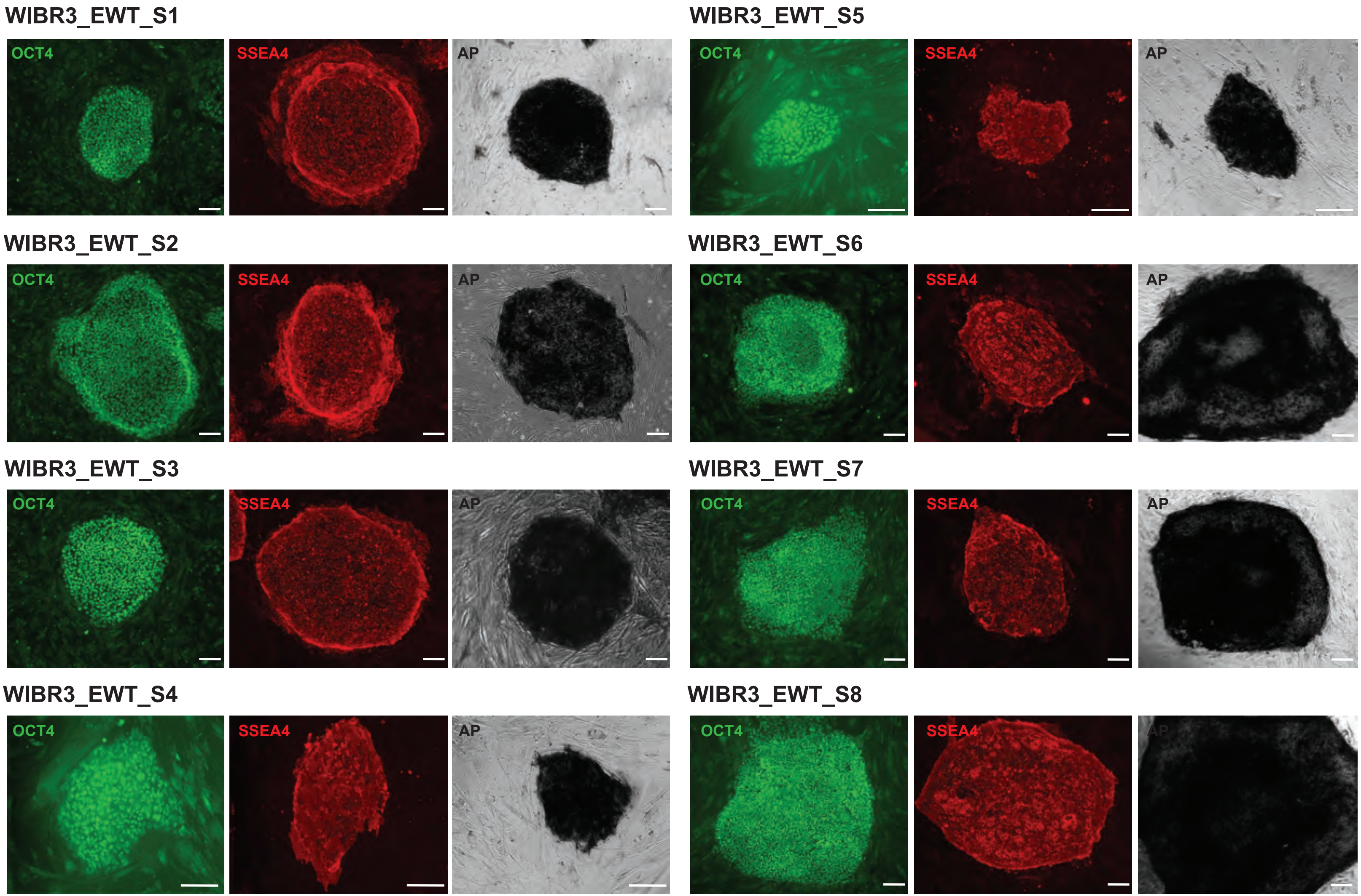

Supplemental Figure 7. SNCA, Busquets et al.

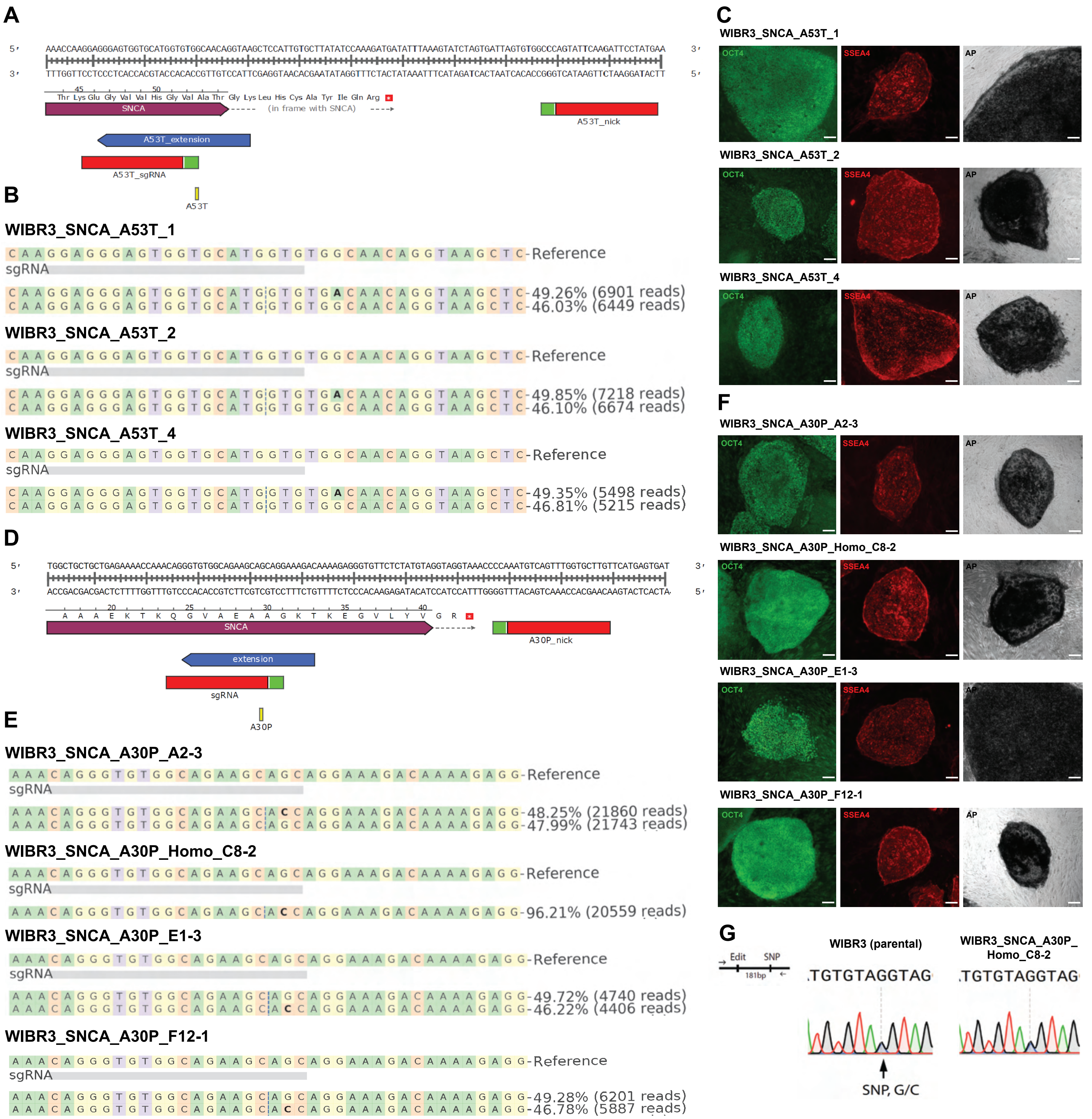

Supplemental Figure 8, PRKN, Busquets et al.

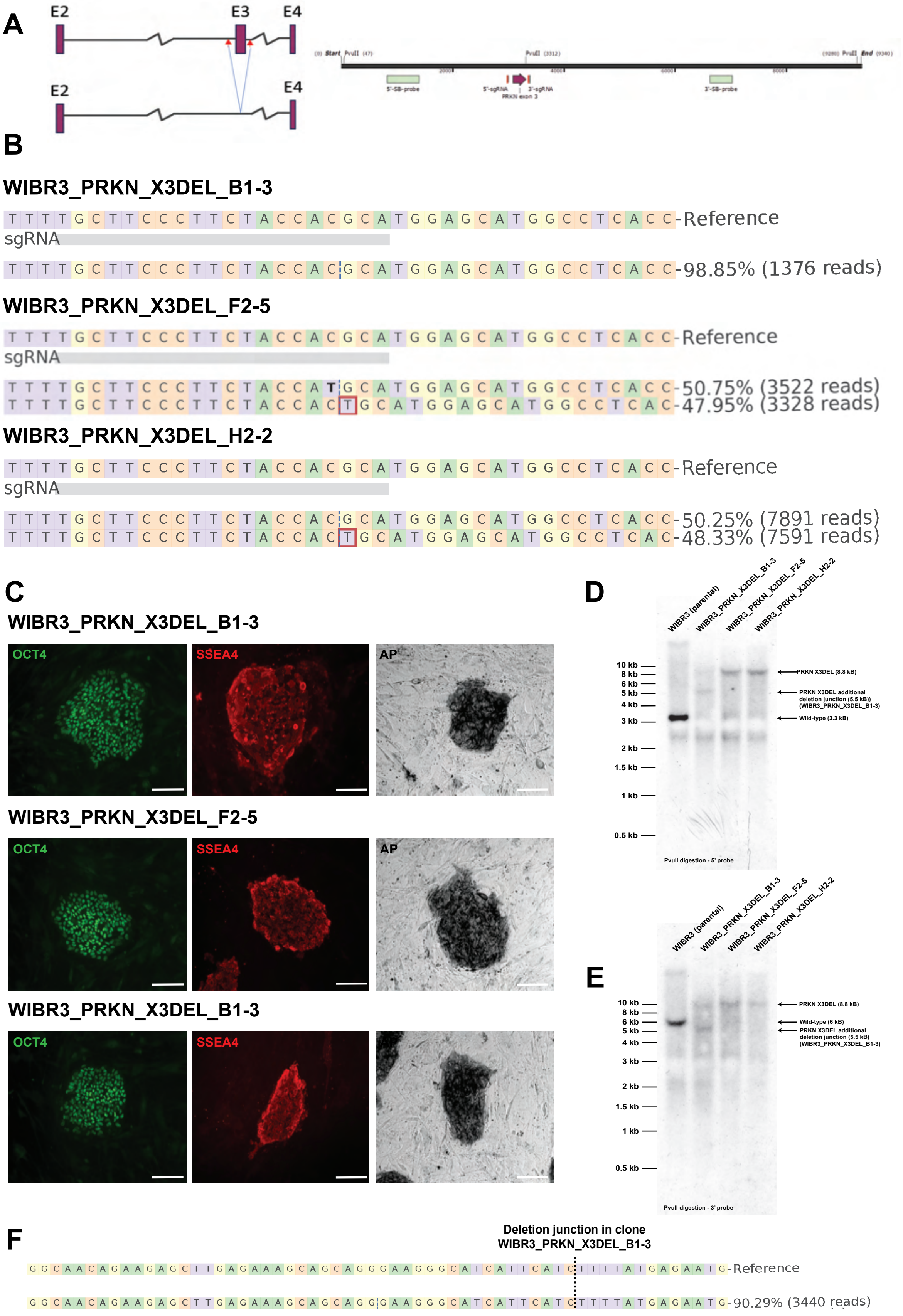

Supplemental Figure 9. PINK1, Busquets et al.

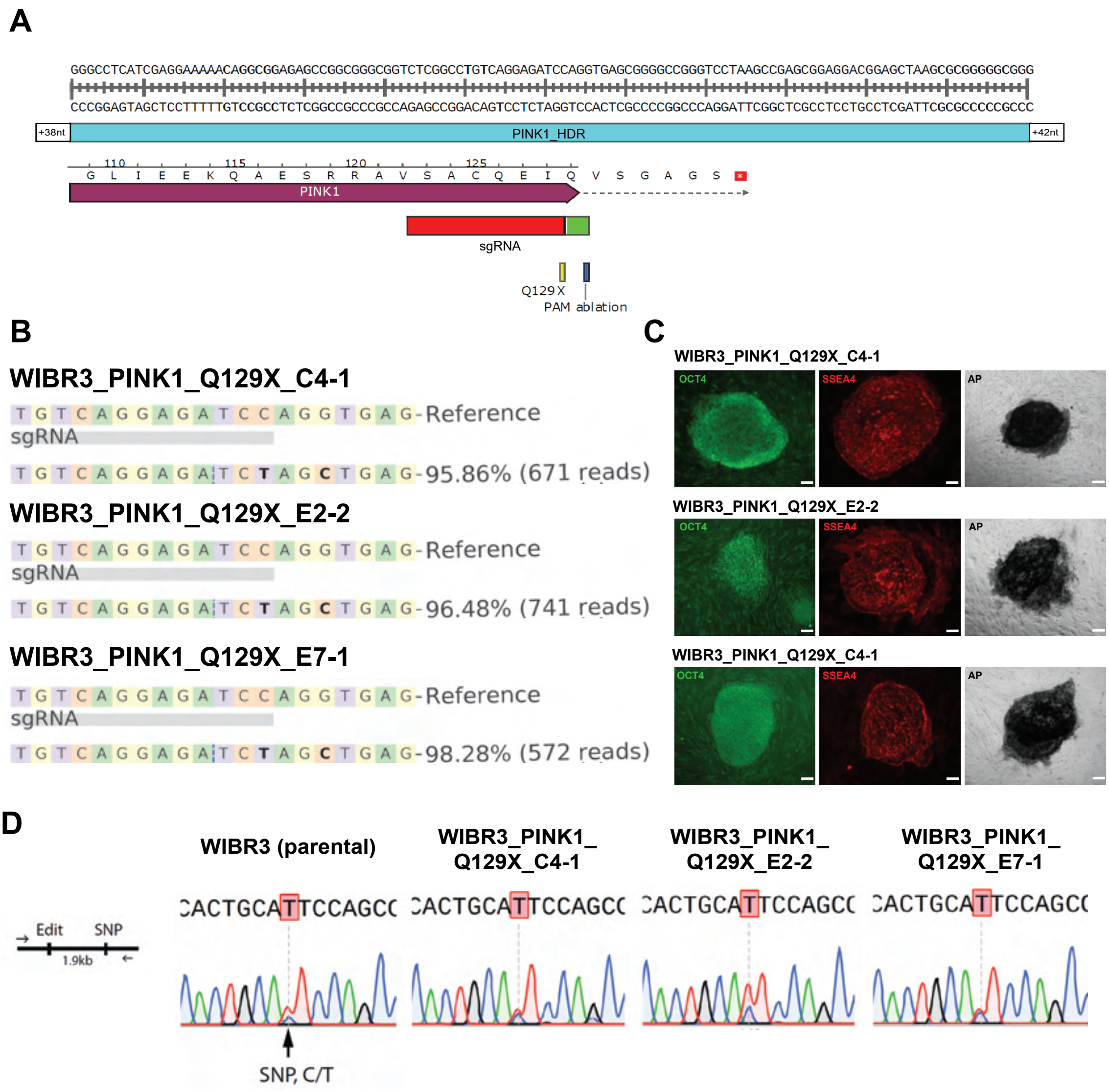

Supplemental Figure 10, DJ1. Busquets et al.

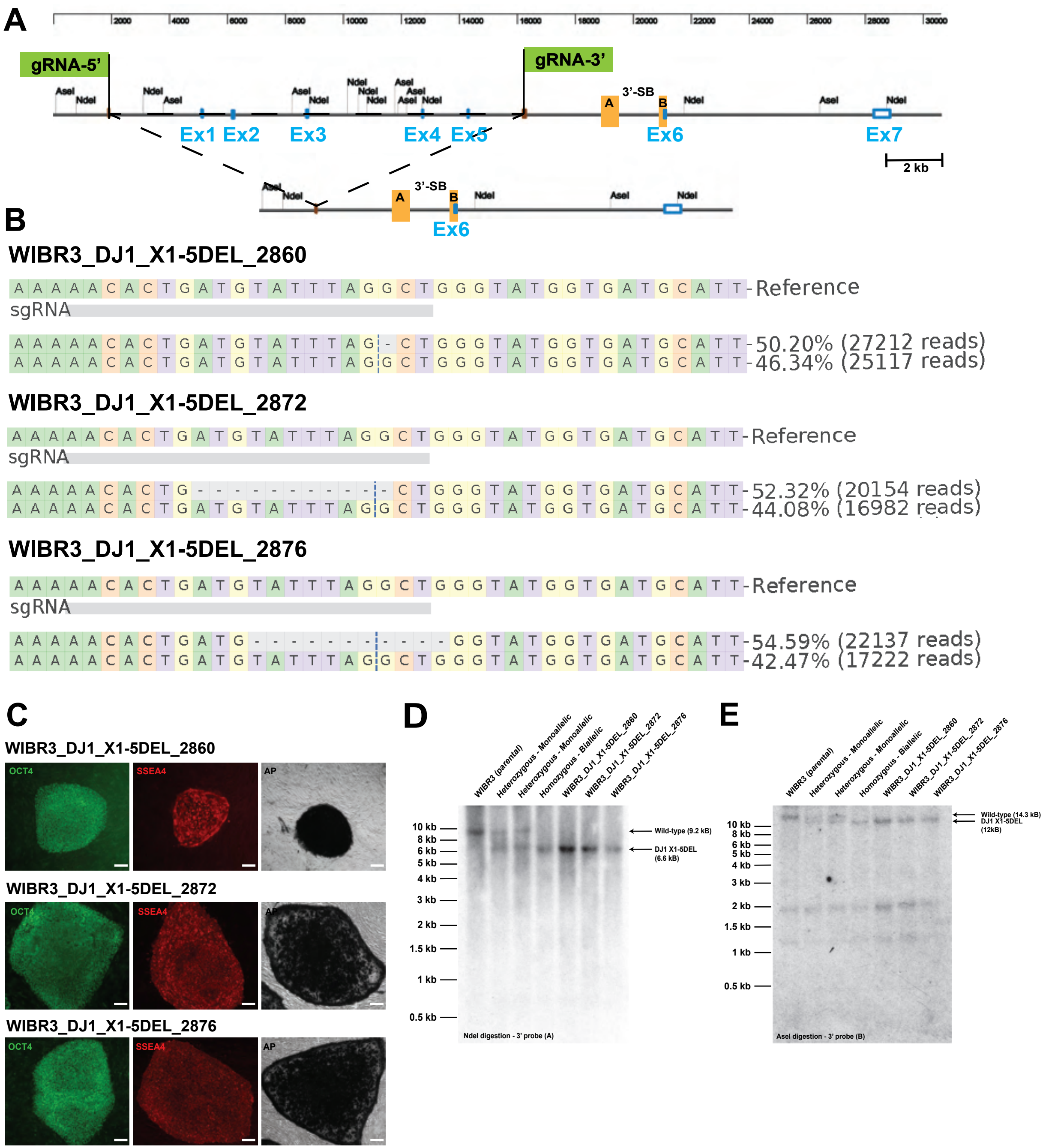

Supplemental Figure 11. LRRK2, Busquets et al.

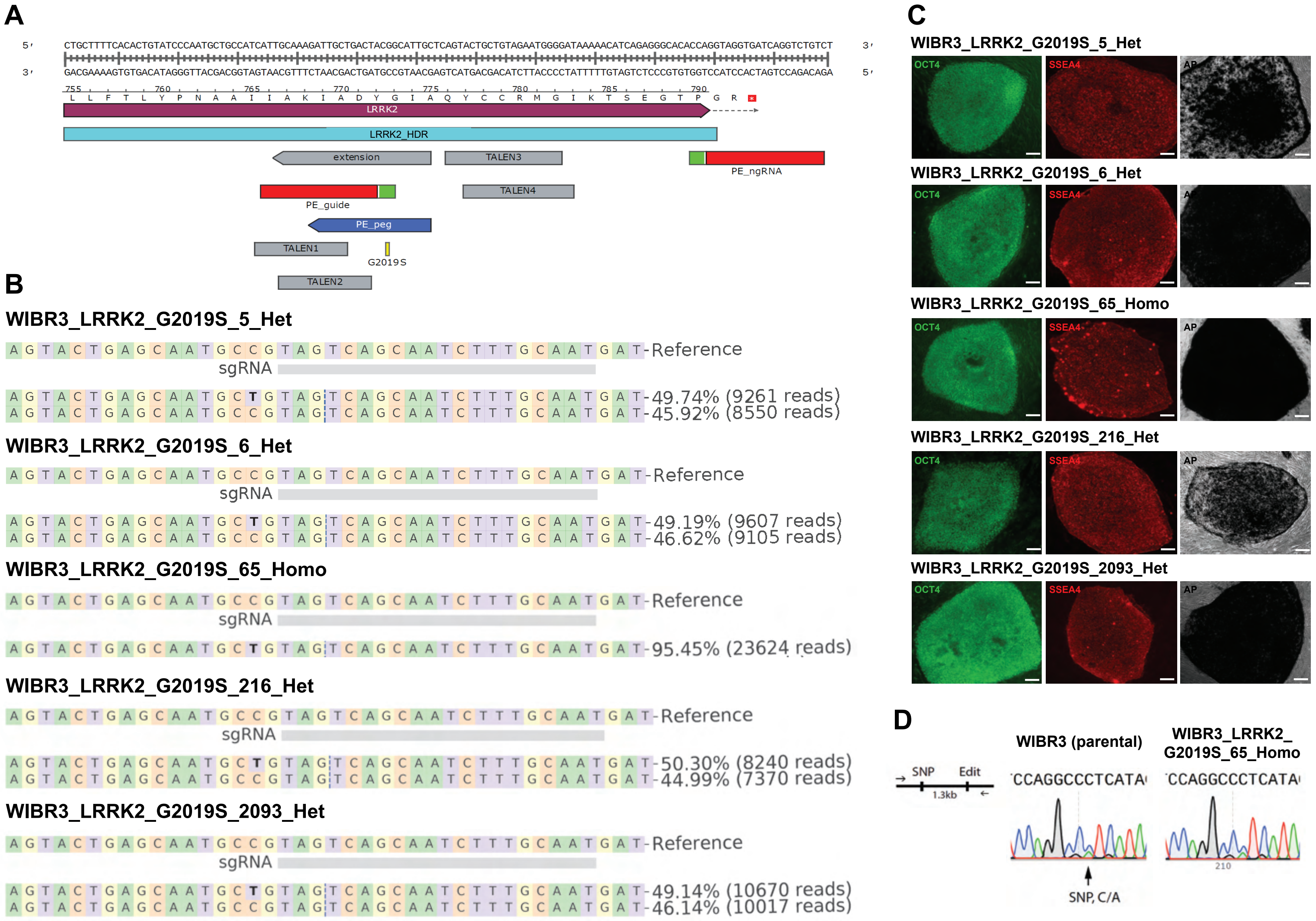

Supplemental Figure 12. ATP13A2, Busquets et al.

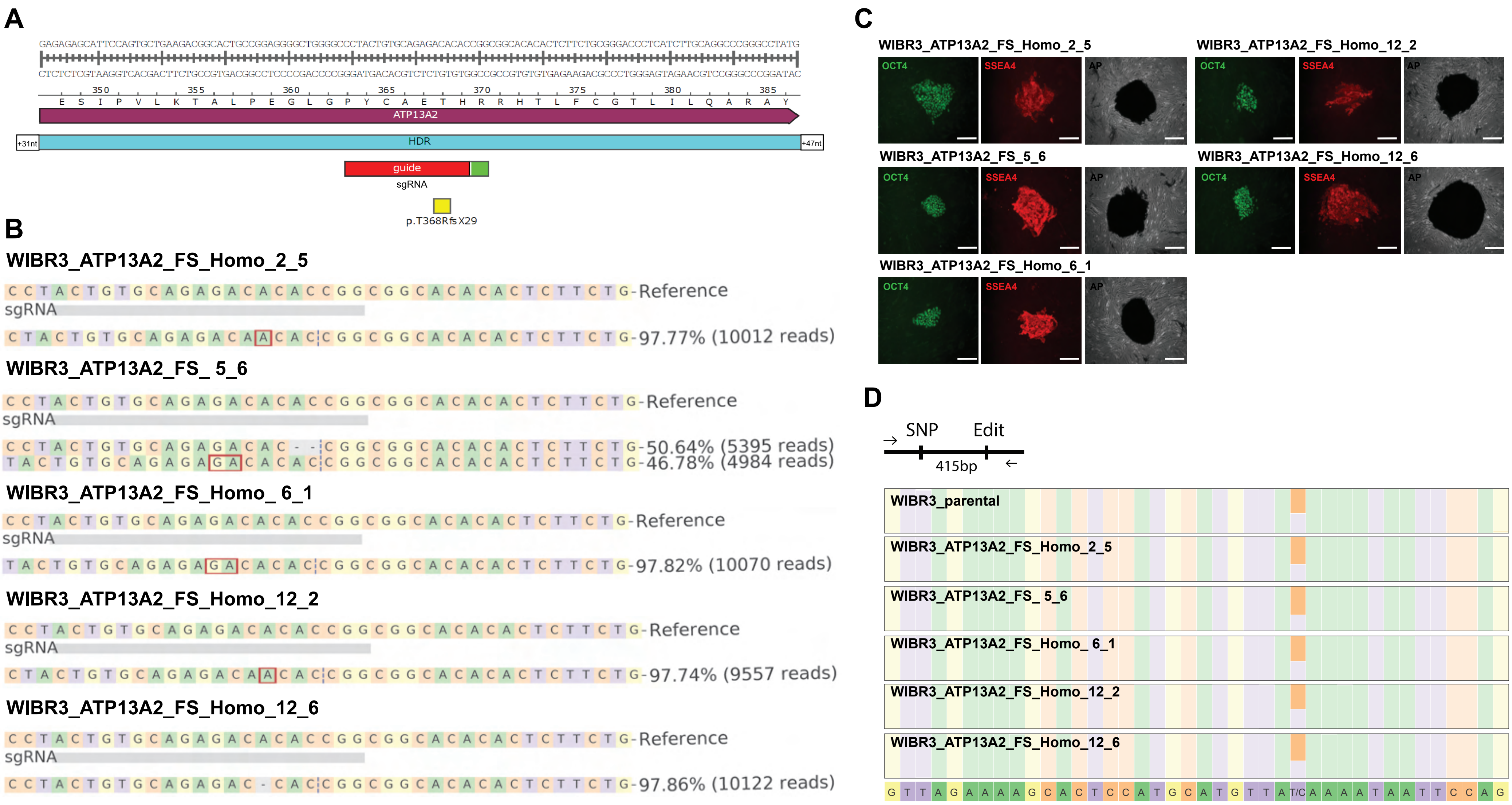

Supplemental Figure 13. FBXO7, Busquets et al.

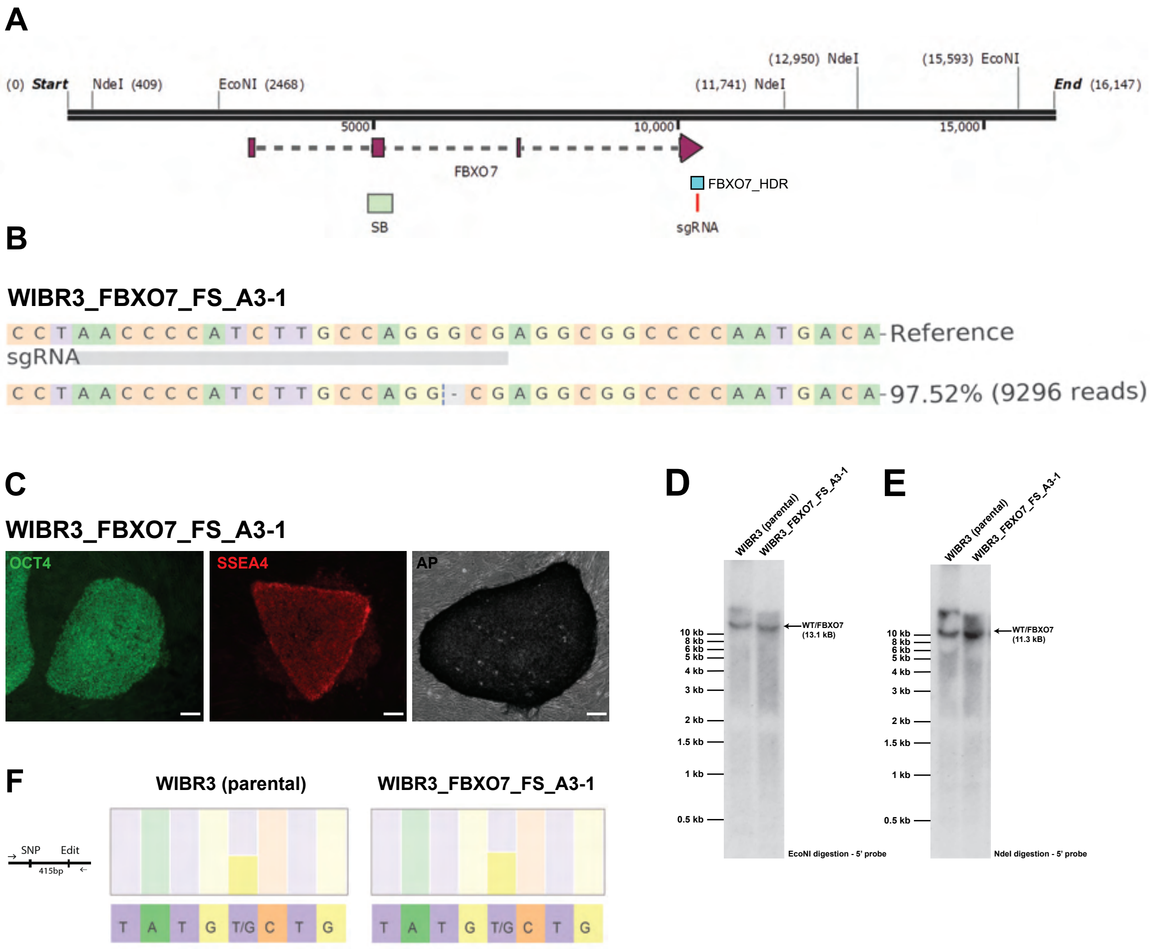

Supplemental Figure 14. DNAJC6, Busquets et al.

A

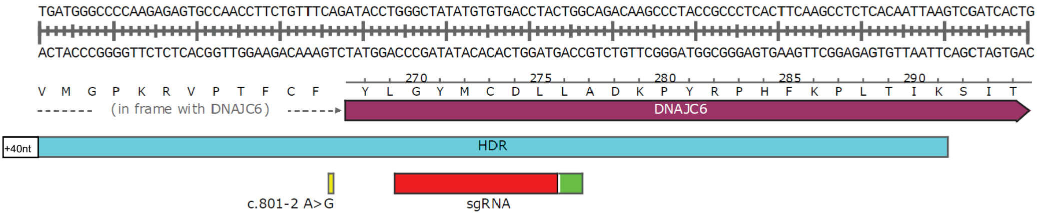

B

WIBR3_DNAJC6_c.801-2 A>G+FS/FS_G12-2

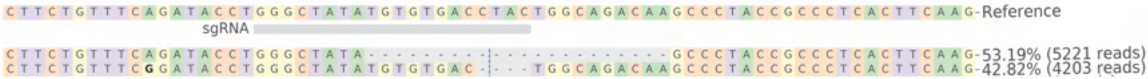

WIBR3_DNAJC6_FS/FS_H10-1

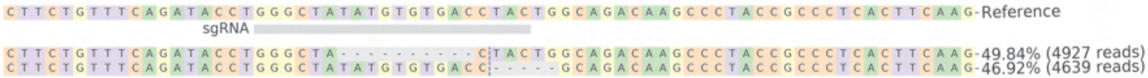

C

WIBR3_DNAJC6_c.801-2 A>G+FS/FS_G12-2

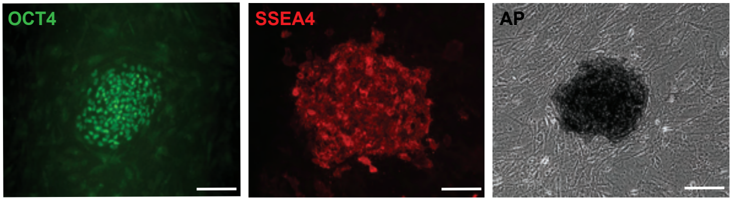

WIBR3_DNAJC6_FS/FS_H10-1

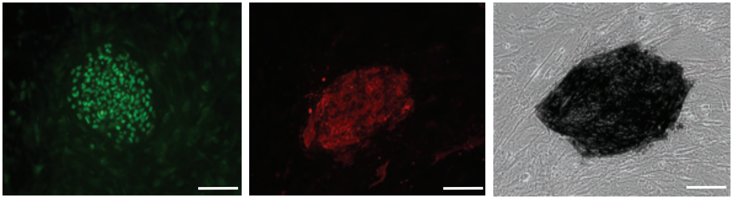

Supplemental Figure 15. SYNJ1, Busquets et al.

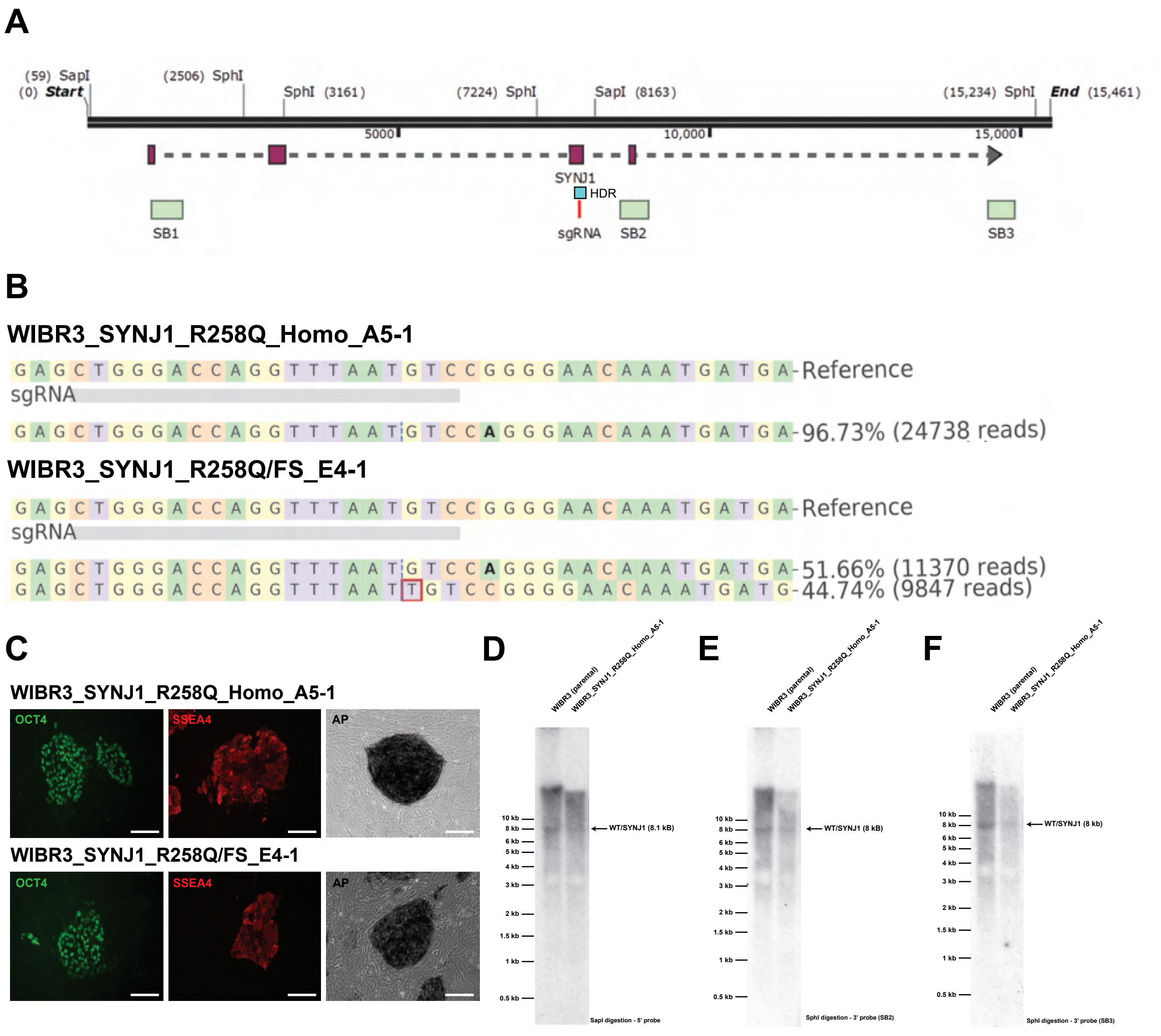

(which was not certified by peer review) is the author/funder, who has granted bioRxiv a license to display the preprint in perpetuity. It is made available under aCC-BY 4.0 International license.

Supplemental Figure 16. VPS13c, Busquets et al.

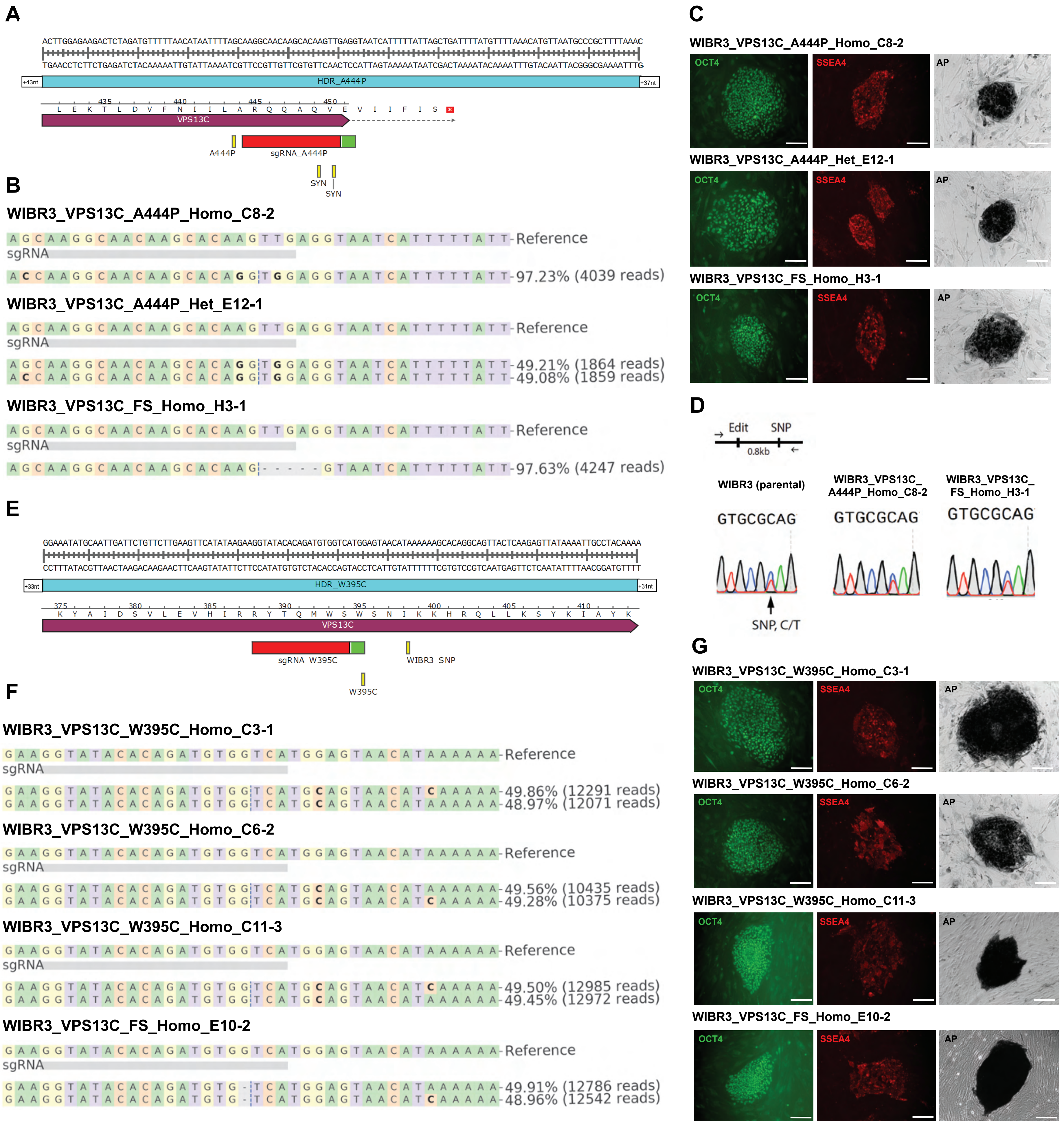

Supplemental Figure 17. GBA, Busquets et al.

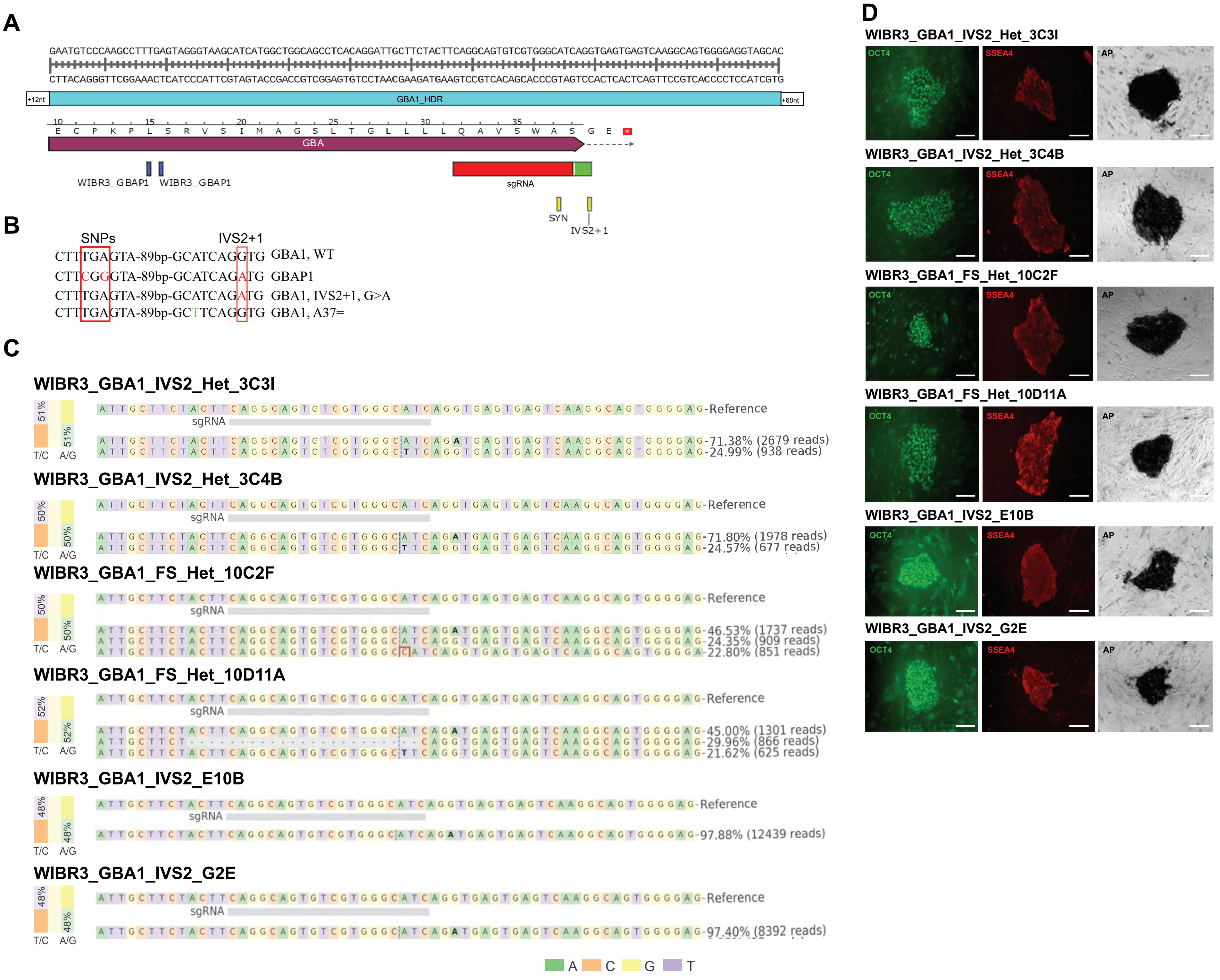

Supplement: Supplementary file 1 [file NIHPP2024.02.12.579917V1-supplement-1.pdf]
